# Forecasting the Price of Gold with Integrated Media Sentiment—A Prediction Framework Based on Online News Sentiment Mining with CNN-QRLSTM

**DOI:** 10.3390/e28030271

**Published:** 2026-02-28

**Authors:** Yu Ji, Xinyue Lei, Lining Zhang, Jiani Heng, Jianwei Fan

**Affiliations:** 1 School of Mathematics and Statistics, Beijing Technology and Business University, Beijing 100048, China; 2201013106@st.btbu.edu.cn (Y.J.); 2201013108@st.btbu.edu.cn (X.L.); 2 School of Economics, Beijing Technology and Business University, Beijing 100048, China; 2401050235@st.btbu.edu.cn; 3 School of Transportation, Southeast University, Nanjing 211189, China; fanjianwei@seu.edu.cn

**Keywords:** gold price forecast, Hurst index, EEMD, CNN-QRLSTM, online news emotional poles *BI*, information theory, entropy, mutual information

## Abstract

Accurate gold price forecasting is crucial for economic stability and investment decision-making. In order to improve the accuracy of gold price prediction and quantify the uncertainty of gold price fluctuation, this paper proposes a hybrid model (CNN-QRLSTM) that integrates convolutional neural network (CNN) and quantile regression long- and short-term memory network (QRLSTM) and innovatively introduces news text data to quantify the media sentiment. We combine EEMD with the Hurst index to remove white noise from the original signal, and the processed data is used as the input layer of the prediction model. Furthermore, to demonstrate the impact of news sentiment on gold prices, this paper employs entropy measurement methods based on information theory to quantify the uncertainty and information content embedded within processed gold price sequences and derived sentiment indicators. The mutual information (MI) algorithm, based on information entropy, captures the nonlinear correlations between financial keywords and market sentiment. It constructs a financial sentiment lexicon (covering keywords such as economic policies and geopolitical conflicts), combines semantic rules with context-weighted strategies, calculates sentiment scores for news texts, and generates daily aggregated media sentiment indicators. This entropy-based perception method not only enhances the interpretability of emotion-driven fluctuations but also provides a theoretical foundation for reducing prediction uncertainty through multi-source data fusion. The experiment uses 2022–2025 daily London gold spot price data, Shanghai Gold Exchange gold price data, and the same period of Gold Investment Network gold market news to carry out the study. The empirical study shows that the synergy of multi-source data fusion and the quantile regression mechanism can improve the accuracy of gold price prediction and the new paradigm of risk interpretation while providing theoretical support for the formulation of quantitative investment strategies.

## 1. Introduction

### 1.1. Background and Significance of the Study

Gold serves as a commodity, currency, and safe-haven asset in global markets. Its price fluctuations serve as an economic barometer and directly impact inflation hedging, exchange rate risk management, and portfolio allocation [1]. Research shows that gold plays a crucial role in clean energy development [2]. However, gold prices are influenced by a complex interplay of factors, such as fluctuating real interest rates, shifts in the U.S. dollar index, geopolitical tensions like trade wars, macroeconomic policy changes such as central bank interest rate adjustments, and prevailing market sentiment. These factors create complex gold price time series with nonlinearity, high noise, and structural breaks. Traditional forecasting models struggle with insufficient accuracy and poor robustness, particularly in adapting to the volatile and unpredictable nature of gold price movements [3].

In recent years, with the proliferation of digital media and real-time data sources, vast volumes of news texts have become a crucial medium for reflecting market sentiment and expectations. Existing research confirms that investor sentiment and media bias significantly influence short-term fluctuations in gold prices. However, effectively extracting domain-specific sentiment signals from unstructured text and deeply integrating them with quantitative financial data remains a frontier challenge in current research. Furthermore, most predictive models provide only point estimates, making it difficult to quantify prediction uncertainty and failing to meet the demand for probabilistic decision support in risk management practices. Coupled with the rapid development of the Internet, the volume and types of data continue to expand, so accurately predicting the price of gold has become a serious and urgent problem. From an information-theoretic perspective, gold price volatility embodies a high degree of entropy—a measure of market uncertainty and information randomness. Effectively quantifying and reducing this entropy through data processing and feature extraction is key to improving forecast robustness.

### 1.2. Literature Review

Effective forecasting of the price of gold is of great significance not only for the formulation of strategies to cope with risks and the maintenance of economic stability but also for its far-reaching impact on the stable development of international financial markets. In recent years, domestic and foreign research on the price of gold is mainly divided into two categories: one is to explore the influencing factors of the price of gold, and the other is committed to proposing a prediction model for the price of gold [4,5]. This part will elaborate on the research results of the literature from these two aspects, respectively, summarize the important factors affecting the price of gold, and summarize the research ideas and forecasting methods of the gold forecasting model.

#### 1.2.1. Factors Affecting the Price of Gold

Most of the current research on the factors influencing the price of gold has centered on macroeconomics as a central factor. Haque (2015) employed Dickey–Fuller and DF-GLS unit root tests to demonstrate a positive correlation between gold prices and the Australian dollar to US dollar (AUD/USD) exchange rate [6]. Huang (2010) and others analyzed the correlation between the dollar index and the price of gold, and the results showed that there was a negative correlation between the two before, and there is no cointegration relationship, which is affected by a variety of factors [7]. Xie (2010) analyzed the correlation between the US dollar and the world gold price from a long-term historical perspective to establish a cointegration equation [8]. The conclusion shows that there is a little bit of cointegration between the world gold price and the US dollar. In Dan (2009), with the data of gold price and domestic inflation from 1996 to 2007 in China, the correlation between them is analyzed with the Phillips expanding curve equation and the method of least squares estimation [9]. The results show that the price of gold plays a role in forecasting the inflation. Therefore, the price of gold can be used as a reference to indicate the economic trends and the changes in the inflation fluctuations. Zhu (2018) conducted an event study analyzing the impact of monetary policy from four major central banks on the dollar gold price [10]. They concluded that the Federal Reserve and the European Central Bank exerted significant influence on gold prices, while the monetary policies of the Bank of England and the Bank of Japan had no discernible effect on gold prices.

In addition to this, some other factors influencing the price of gold have also been suggested by scholars, mainly real interest rates, geopolitical risks, commodity prices, and weather factors, as well as economic policy uncertainty and investor sentiment. Zhou (2025) investigated the relationship between geopolitical risk (GPR) and gold price bubbles [11]. The results indicate a significant relationship between GPR and gold price bubbles, particularly with GPRA, which exerts a stronger influence than GPRT does. Apergis (2019) employed a Bayesian Markov Switching Vector Error Correction Model (VECM) to uncover a positive correlation between gold prices and real interest rates, demonstrating that gold can hedge against fluctuations in real interest rates during economic recessions [12]. Kanjilal (2017) employed a dual-mechanism threshold vector correction model to identify distinct dynamics in gold and crude oil across long-term and short-term states following the 2008 global crisis [13]. Their findings indicate that the relationship between gold and oil prices is nonlinear and asymmetric. Zhang (2010) examined the price volatility of two markets, oil and gold, and analyzed their cointegration and causality, and the results of the study showed that the price of crude oil and the price of gold were in agreement, and the positive correlation coefficient was significant [14]. Zhu (2023) re-examined the potential driving impact of climate risks on gold price volatility using predictive modeling and seasonal-trend decomposition (STL) methods, observing the utility benefits of such risks [15]. They found that gold price fluctuations are negatively correlated with physical risks. Soni (2023) employed a wavelet-based approach to examine the dynamic relationship between Indian economic policy (EPU) uncertainty and oil, stocks, and gold [16]. Their findings indicate that in the medium to short term, gold prices exhibit a positive correlation coefficient and covariance with EPU, though the impact is not statistically significant; from a long-term perspective, EPU has a positive impact on gold prices. Luo (2022) utilized an infinite Hidden Markov (IHM) transformation model within a Heterogeneous Autoregressive (HAR) framework to accommodate structural breaks in the data and, for the first time in the literature, use a variety of sentiment indicators representing the speculative and hedging tendencies of investors in these markets as predictors in forecasting models [17]. The results highlight the predictive role of investor sentiment-related factors in improving the forecast accuracy of volatility dynamics in commodities with the potential to also yield economic gains for investors in these markets.

Table 1 summarizes the factors influencing gold prices and their research methodologies as identified in the aforementioned literature.

#### 1.2.2. Gold Price Forecasting Model

In order to improve the accuracy of gold price forecasting, a large number of domestic and foreign financial and statistical scholars have proposed new research directions and forecasting methods. The first group of studies focuses on modeling from traditional statistics. Ismail (2009) constructed a multiple regression model to forecast future trends in gold prices based on influencing factors such as inflation rates, exchange rates, and money supply [18]. Amina (2015) employed a bivariate vector autoregression-generalized autoregressive conditional heteroskedasticity (VAR-GARCH) model to examine the dynamic returns and forecasting capabilities of China’s gold and stock markets, thereby assessing the diversification and hedging effectiveness of Chinese gold [19]. Although the above methods can achieve good results when dealing with linear relationships, they often suffer from overfitting or insufficient prediction accuracy when confronted with the nonlinearity and high volatility of financial markets.

The second type of research is the prediction of gold prices based on machine learning algorithms. Li (2024) developed a novel model by integrating a BP neural network with ensemble empirical mode decomposition [20]. Compared to the original BP neural network, this approach reduces the impact of white noise and enhances the model’s robustness. Hadavandi (2010) proposed a particle swarm optimization (PSO)-based time series model for gold price forecasting, which uses the PSO algorithm for parameter estimation and applies the model to the daily observation of gold prices [21]. The model is tested to be able to cope with the volatility of the gold price time series. You (2025) enhanced prediction accuracy by extracting data features through a combination of convolutional neural networks (CNN) and long short-term memory (LSTM) models [22]. While this type of machine learning has largely improved the accuracy of gold price prediction, its dataset is mostly homogeneous, and its sensitivity to parameter settings needs to be improved.

Therefore, it has become a mainstream approach to improve the predictive performance of traditional statistical models by combining them with machine learning. Poor (2024) introduced unstructured data through text analysis to construct a CNN-based gold price prediction model, demonstrating the significance of multi-source data-driven models and providing investors and financial institutions with a high-precision decision support tool [4]. Solikhun (2025) used a quantum perceptron algorithm to predict global gold prices, utilizing it to improve the efficiency and performance of neural network models [23]. Quantum computers can perform multiple computations simultaneously, allowing them to solve problems that are difficult for classical computers to solve. Nallamothul (2024) combined the skewness–kurtosis generalized autoregressive conditional heteroskedasticity (SKGARCH) model with LSTM to address the inadequacies of traditional methods in fully accounting for volatility information and non-normal distribution characteristics [24]. In Guo (2025), the Complete Ensemble Empirical Mode Decomposition with Adaptive Noise (CEEMDAN) is employed to decompose a residual term containing complex information following the variational modal decomposition (VMD) and an extreme gradient boosting tree (XGBoost) optimized by the Whale Optimization Algorithm (WOA) is combined to construct the VMD-RES.-CEEMDAN-WOA-XGBoost model [25]. It is shown that the combined prediction model has superior performance compared to the other comparative prediction models evaluated. Bhavana (2025) introduces a multi-objective optimization framework that utilizes Pareto alpha cutting techniques to evaluate and enhance gold price forecasting models [26]. They employed three distinct models, the Autoregressive Distributed Lag (ARDL) model, a stochastic model, and the Autoregressive Integrated Moving Average (ARIMA) model, to capture the underlying dynamics of gold price fluctuations influenced by macroeconomic factors. By applying the alpha-cut technique, they filtered out less optimal models, retaining only those that met a predefined level of acceptability across all criteria. The results show that ARDL achieves excellent accuracy and goodness-of-fit, while the stochastic model exhibits robust stability. Gijy (2025) proposes a hybrid Markov Weighted Fuzzy Kernel Time Series framework for gold price prediction, together with Red Piranha Walrus Optimization (MWFKTS-RPWO) [27]. It provides an optimal balance between computational efficiency and accuracy compared to existing methods. Wu (2025) proposed an improved brain-inspired neural network based on the GELU function and residual connections for predicting the next-day stock prices of power companies [28]. By utilizing residual connections to transmit information between shallow and deep layers, the network fully leverages information to mine deep hidden features. Chen (2025) proposed a DROI framework incorporating econometric breakpoint tests to evaluate the predictive performance of Spanish electricity price data across different time periods [29]. This approach effectively addressed the inherent complexities of electricity prices, such as seasonality, high volatility, and non-stationarity. Che (2025) proposed a multivariate method incorporating a genetic algorithm-based feature selection framework (GAFSF) and double bidirectional gated recurrent unit (DBiGRU) to create such a model [30]. It employs multi-temporal and spatial characteristics for wind speed modeling to enhance information acquisition and complex pattern analysis.

Table 2 summarizes the various models used to forecast gold prices in the aforementioned literature, along with their research methodologies.

#### 1.2.3. Information-Theoretic Approaches in Financial Forecasting

Beyond statistical and machine learning models, information theory offers a fundamental framework for quantifying uncertainty, information flow, and feature relevance in financial time series. Entropy, as a core concept, measures the unpredictability of price movements and the information content of market signals. Recent studies have incorporated entropy-based methods (e.g., approximate entropy, sample entropy) to characterize market complexity and detect regime shifts. Furthermore, Mutual Information (MI), a measure of nonlinear dependence derived from entropy, has been applied in feature selection and lexicon expansion for sentiment analysis, capturing contextual associations beyond linear correlation. Our work integrates these principles by using MI for sentiment dictionary construction and employing entropy-aware evaluation to assess uncertainty reduction in gold price forecasts.

#### 1.2.4. Existing Model Flaws and the Innovation of This Paper

To construct a more robust, precise, and interpretable gold price forecasting framework, we first systematically reviewed the limitations of existing mainstream methods and, based on this analysis, proposed the innovative contributions of this study.

##### Limitations of Existing Models and Methods

(1) Noise interference and information redundancy at the data level: Original financial time series exhibit non-stationarity and high noise characteristics. Traditional preprocessing methods (such as simple filtering or wavelet transforms) often lack adaptive capabilities when distinguishing genuine market signals from random noise, potentially leading to the loss of useful high-frequency information or residual noise. This introduces sources of uncertainty into subsequent forecasting models.

(2) The singularity of the model structure and the inadequacy of risk quantification: Mainstream forecasting models can be divided into two categories. First, traditional statistical models (such as ARIMA and GARCH) can provide interval forecasts but struggle to capture complex nonlinear relationships. Second, machine learning models (such as LSTM and CNN), while adept at capturing nonlinear patterns, cannot quantify prediction uncertainty through their point-prediction outputs, making them ill-suited for risk management requirements. Simple model combinations fail to achieve deep coordination among local feature extraction, modeling long-term dependencies, and probabilistic output.

(3) The shallow integration of multi-source data is particularly evident in the realm of sentiment analysis: Most studies merely incorporate macroeconomic indicators as supplementary inputs, while the utilization of alternative data (such as news texts) remains relatively superficial. Specifically, sentiment fusion prediction models such as Sentiment LSTM or emotion-enhanced attention mechanisms generally exhibit limitations. First, sentiment representation is limited, heavily relying on pre-built generic sentiment dictionaries without dynamic expansion tailored to the financial sector. This makes it difficult to capture sentiment semantics within market-specific contexts such as “safe-haven” and “policy uncertainty.” Second, the temporal alignment is coarse, with sentiment indicators often aggregated at daily intervals. When synchronizing with price sequences, the specific timing of news releases and the lag effects of market reactions are not sufficiently accounted for. This may cause sentiment signals to lag or lead, introducing misleading correlations. Third, the model integration remains superficial. Emotional features are typically fed directly into recurrent neural networks as supplementary input vectors without undergoing more refined local semantic structure extraction through sophisticated architectures. Furthermore, they are not synergistically optimized with quantifiable uncertainty mechanisms, thereby limiting the potential of emotional information to enhance prediction accuracy and risk insights.

(4) The Static Nature and Domain Barriers in Emotion Indicator Construction: Sentiment analysis methods based on fixed, general-purpose dictionaries struggle to adapt to the dynamic context of financial texts and fail to recognize the sentiment weighting of domain-specific keywords. This results in constructed sentiment metrics that deviate from actual market sentiment.

##### Innovative Contributions of This Paper

To address the aforementioned limitations, this paper introduces systematic innovations across four dimensions: data processing, model architecture, information fusion, and emotion quantification.

(1) To overcome the poor adaptability of traditional denoising methods, this paper proposes an advanced denoising strategy based on EEMD. After decomposing into multi-scale intrinsic modal functions (IMFs), the approach does not simply eliminate high-frequency components. Instead, it introduces a dual criterion combining the Hurst exponent and sample entropy. An IMF is only classified as predominantly random noise and excluded when it simultaneously exhibits a low Hurst index indicating anti-persistence and high sample entropy indicating high randomness. This method more precisely separates noise from valid signals by analyzing both statistical characteristics and information structure, providing cleaner and more stable inputs for subsequent predictions.

(2) To unify the nonlinear fitting capability and uncertainty quantification capability of the model, this paper proposes a novel hybrid deep learning framework, CNN-QRLSTM. Within this model, CNN provides local perception and parameter sharing capabilities, effectively capturing local patterns in input sequences while reducing model complexity. QR performs interval estimation across different quantiles and employs a quantile loss function to weight cross-quantile prediction errors, thereby optimizing forecast outcomes. As a core component, LSTM captures long-term dependencies within sequences and demonstrates memory capabilities. This method excels in nonlinear fitting and is suitable for forecasting both short-term and long-term gold prices characterized by inherent volatility.

(3) This paper constructs a hierarchical multi-source data input system. In terms of structured data, the system incorporates eight major categories of factors, including macroeconomics, monetary policy, commodities, and climate risk. In the utilization of unstructured data, this study proposes a solution fundamentally distinct from existing sentiment fusion models. (1) Domain-adaptive sentiment construction dynamically expands the sentiment lexicon from financial news based on mutual information (MI), ensuring sentiment representations remain highly relevant to the gold market context and resolving semantic disconnect issues inherent in generic dictionaries. (2) Through multi-level feature collaboration, the CNN-QRLSTM framework establishes a progressive architecture comprising “local perception (CNN)—temporal modeling (LSTM)—risk assessment (QR).” (3) Percentile-aware emotion fusion, where emotion indicators not only serve as inputs for point predictions but also directly participate in percentile loss calculations.

(4) To achieve precise and robust domain sentiment representation, this paper proposes a method for constructing and expanding a financial sentiment lexicon based on mutual information (MI) from information theory, accompanied by rigorous experimental design to mitigate risks. Starting from seed words, the method dynamically identifies and incorporates sentiment terms with strong domain semantic associations by calculating MI values between candidate words and sets of positive/negative seed words across vast financial news. Furthermore, when calculating the daily sentiment polarity index (BI) and training the model, strict adherence to temporal sequencing is maintained. Only news from the current day and historical periods are utilized, with a one-period lag applied to the model input. This approach effectively mitigates risks of information leakage and overfitting.

In summary, the four core innovations of this study form a one-to-one solution relationship with the aforementioned four major limitations. Together, they constitute a complete, coherent, and logically consistent predictive framework innovation system—spanning data cleansing, model design, information fusion, and feature engineering—aimed at systematically enhancing the accuracy, robustness, and decision-making utility of gold price forecasting.

The remainder of this paper is organized as follows. In Section 2, the basic principles of the proposed model and correction method are introduced, respectively. Section 3 outlines the dataset partitioning and presents experimental results for all models, including data processing outcomes, price prediction results, and metric validation findings. In Section 4, the model’s capabilities in interval coverage, time dependency, and extreme event prediction are examined. In Section 5, the experimental results are summarized and future research prospects are discussed.

## 2. Methodology

### 2.1. EEMD–Hurst–Entropy Model Theory

#### 2.1.1. EEMD Model

Integrated Empirical Modal Decomposition (EEMD) is an improved method of Empirical Modal Decomposition (EMD) that suppresses the defects of EMD mode mixing by adding white noise. The core idea of EEMD is to superimpose different Gaussian white noise sequences of the same amplitude on the original signal to change the distribution of the signal extrema and obtain the upper and lower envelopes that conform to the characteristics of the signal, and then obtain each intrinsic modal function (IMF) by EMD of the superimposed signals several times, and then perform overall averaging of the IMF results to offset the added white noise, so as to effectively inhibit modal aliasing and avoid signal decomposition distortion. In this way, the generation of modal aliasing can be effectively suppressed, and signal decomposition distortion can be avoided.

First for the original signal, add Gaussian white noise,

(1) $$x_{j}(t)=x(t)+\varepsilon \cdot \omega_{j}(t),j=1,2,\ldots ,M,$$

where $\omega_{j}(t)$ is a Gaussian white noise added for the first time. *M* is the total number of integrations. ε is the noise amplitude factor.

Perform EMD on the signal $x_{j}(t)$ after each noise addition. Finding all the points of great and small values of the, construct the upper envelope $e_{\max}(t)$ and lower envelope $e_{\min}(t)$ of the signal using cubic spline interpolation, and compute the local mean,

(2) $$m(t)=\frac{e_{\max}(t)+e_{\min}(t)}{2},$$

Extract IMF for iterative screening,

(3) $$h_{i}(t)=h_{i-1}(t)-m_{i-1}(t),$$

Until $h_{i}(t)$ satisfies the IMF conditions. The standard deviation (*SD*) is typically used as the stopping criterion.

(4) $$\left\{\begin{array}{l} \mathrm{Standard}\;\mathrm{Deviation}\;(\mathrm{SD})\;\mathrm{Guideline}:\;SD=\sum_{t}\frac{{\left|m_{i-1}(t)\right|}^{2}}{h_{i-1}^{2}}<\varepsilon \\ \mathrm{Difference}\;\leq 1:\;\left|N_{extrema}-N_{zero-cros\sin gs}\right|\leq 1 \end{array}\right.$$

A series of intrinsic modal functions (IMFs) and a residual function are obtained,

(5) $$x_{j}(t)=\sum_{j=1}^{M}c_{j,i}(t)+r_{j,n}(t),$$

where $c_{j,i}(t)$ is the i-th IMF of the j-th decomposition. $r_{j,n}(t)$ is a residual function.

The same order IMF of all the noise-added signal decomposition results are averaged to obtain the final IMF.

(6) $${\bar{c}}_{i}(t)=\frac{1}{M}\sum_{j=1}^{M}c_{j,i}(t),$$

The final EEMD results in

(7) $$x(t)\approx \sum_{j=1}^{n}{\bar{c}}_{i}(t)+{\bar{r}}_{n}(t),$$

where ${\bar{r}}_{n}(t)$ is the average residual term.

From an information-theoretic perspective, the EEMD process essentially involves a normalized separation of the original signal’s information (or uncertainty). Each intrinsic modal function (IMF) represents a distinct oscillatory pattern at different time scales, each exhibiting varying degrees of information complexity (or randomness). Shannon entropy serves as the classical tool for quantifying this uncertainty. For a discretized IMF component $IMF_{i}$, its entropy value $H\left(IMF_{i}\right)$ can be approximated as

(8) $$H\left(IMF_{i}\right)=-\sum_{k}p_{k}\log_{2}\left(p_{k}\right)$$

where $p_{k}$ represents the probability distribution of $IMF_{i}$’s amplitude after binning. High-frequency, irregular IMF components (such as IMF1 and IMF2) typically correspond to market micro-noise or unpredictable instantaneous shocks, exhibiting high entropy values and disordered information. Conversely, low-frequency, smooth IMF components (such as trend components) possess lower entropy values and stable information structures, representing certain long-term trends. Therefore, incorporating entropy analysis enables more precise identification and removal of IMF components that primarily contribute noise rather than meaningful information.

**Figure 1:**
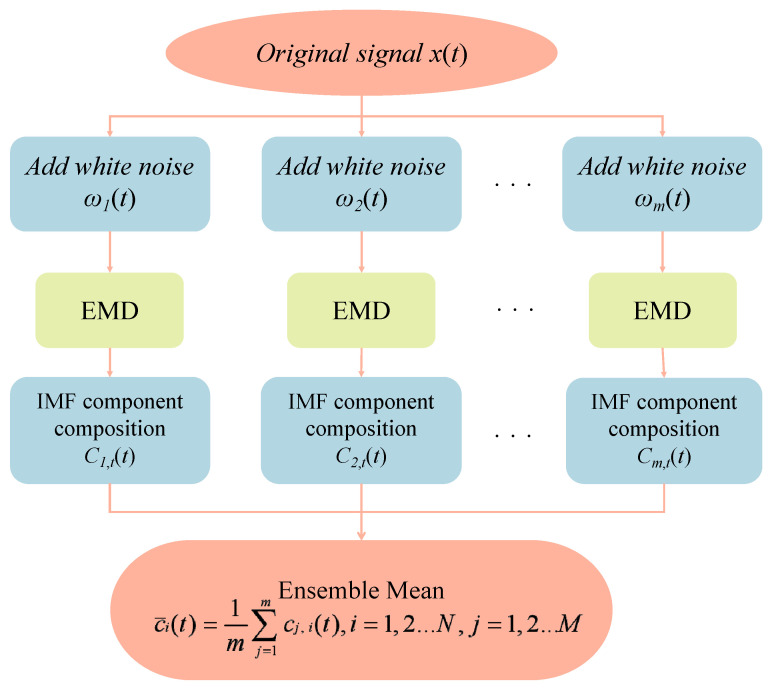
Technical process of EEMD.

Figure 1 illustrates the entire process of data decomposition using the EEMD model.

#### 2.1.2. IMF Selection Based on Hurst Exponents and Information Entropy

To more accurately separate noise from useful information in raw signals, this paper proposes a dual-criterion IMF filtering method based on the Hurst exponent and information entropy. The Hurst exponent measures the persistence of a time series from the perspective of its long-range memory, while information entropy (specifically, sample entropy, as adopted in this paper) quantifies the randomness and irregularity of the sequence from the angle of information complexity. By combining these two complementary metrics, we can scientifically evaluate and screen the IMF components following EEMD at both the statistical characteristics and information structure levels.

##### Introduction of the Hurst Index

The generalized Hurst index describes the scaling behavior of segments with large or small fluctuations. Research indicates that when *h* < 0.5, the time series exhibits anti-persistence; time series show random wandering behavior when h = 0.5; time series are shown to be persistent when *h* > 0.5 [30]. Therefore, when performing EEMD, examine the Hurst exponent of each IMF group and observe the Hurst exponent of the data after removing a specific IMF group, ensuring it remains within the range of [0.5, 0.7].

##### Theory and Computation of Sample Entropy

Sample entropy serves as a robust metric for measuring the complexity and irregularity of time series, particularly well-suited for short data sequences. For an IMF component A of length N, the calculation steps for its sample entropy $SampEn\left(m,r,N\right)$ are as follows:

Construct an m-dimensional vector sequentially,

(9) $$X_{m}(i)=\left[u(i),u(i+1),\ldots ,u(i+m-1)\right],$$

where i=1,2,…,N−m+1.

Define the distance between two distances A and B as the maximum absolute value of the differences between their corresponding elements,

(10) $$d\left[X_{m}(i),X_{m}(j)\right]=\underset{k=0,1,\ldots ,m-1}{\max}\left|u(i+k)-u(j+k)\right|,$$

Given a similarity tolerance *r*, count the number of vector pairs satisfying $d\left[X_{m}(i),X_{m}(j)\right]<r$ and calculate their proportion,

(11) $$B_{i}^{m}(r)=\frac{1}{N-m-1}count\left\{d\left[X_{m}(i),X_{m}(j)\right]<r\right\},$$

Calculate the average,

(12) $$B^{m}(r)=\frac{1}{N-m}\sum_{i=1}^{N-m}B_{i}^{m}(r),$$

Increment dimension m by *m* + 1, repeat the above steps to obtain $B^{m+1}(r)$.

(13) $$SampEn(m,r,N)=-\ln\left[\frac{B^{m+1}(r)}{B^{m}(r)}\right],$$

The higher the entropy value of a sample, the more complex and irregular the sequence becomes, indicating a higher proportion of random noise components. Conversely, the smoother and more regular the sequence, the more defined its information structure becomes.

##### Hurst–Entropy Dual-Criterion Screening Mechanism

After obtaining a series of IMF components through EEMD, we simultaneously calculate the Hurst exponent *H* and sample entropy *SampEn* for each IMF component. Based on information theory and time series analysis principles, we establish the following screening criteria:

Identification of Noise IMFs: For high-frequency IMFs, if both conditions are simultaneously satisfied, (1) H≪0.5; (2) The *SampEn* value is significantly higher than the other components. Then it is determined that the IMF primarily consists of high-frequency random noise and non-predictive interference, and should be removed from the reconstructed signal.

Effective IMF retention: For low-to-mid frequency IMF, if the following conditions are met, (1) H≈0.5 or H>0.5; (2) The *SampEn* value is relatively low. Then it is determined that the IMF carries structural information with predictive value in gold price fluctuations, and should be retained and used for signal reconstruction.

##### Dual-Criteria Screening Process

First, perform EEMD on the original gold price sequence to obtain IMF components arranged from high to low frequency. Next, the Hurst exponent and sample entropy of each IMF are computed in parallel; finally, each IMF is evaluated based on the dual criteria. Those simultaneously satisfying “low Hurst exponent” and “high sample entropy” are identified as noise and removed. The remaining IMF components are reconstructed into a denoised time series, which serves as input for the subsequent CNN-QRLSTM prediction model.

**Figure 2:**
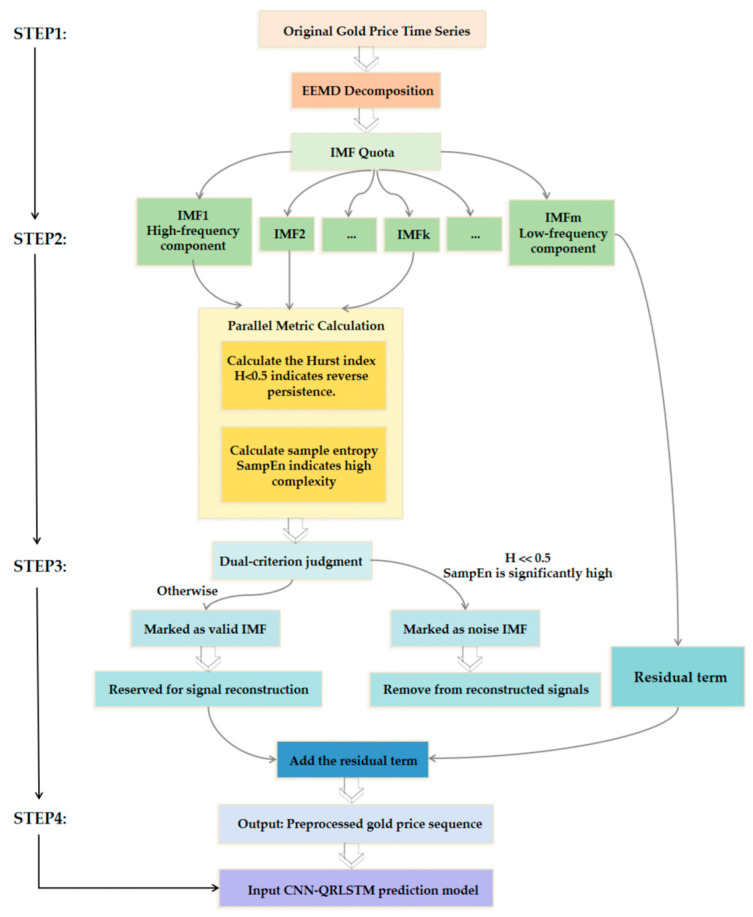
Dual-Criterion IMF Selection Flowchart Based on Hurst Exponent and Sample Entropy.

Figure 2 illustrates the process of filtering sub-sequences after EEMD using the dual criteria of Hurst exponent and sample entropy.

By introducing information entropy (sample entropy) as a complementary metric to the Hurst index, this method achieves quantitative assessment of signal components across two dimensions: “statistical properties” and “information complexity.” This not only provides a more robust theoretical foundation for noise identification but also ensures that data input into prediction models exhibits both stationarity (guaranteed by the Hurst exponent) and low randomness (guaranteed by sample entropy). Consequently, it reduces model learning uncertainty at its source, thereby enhancing prediction accuracy and robustness. Algorithm 1 presents the pseudocode for the EEMD algorithm.
**Algorithm 1.** The EEMD model pseudo-code.
EEMD (Ensemble Empirical Mode Decomposition)Parameters:*N*: Number of ensemble trials (typical range: 100–500)*ϵ*: Noise amplitude ratio (usually 0.1–0.3 of data STD)*IMF_max_*: Maximum number of Intrinsic Mode Functions to extract
Input:
*x*(*t*): Original time series signal (length *T*)*w_n_*(*t*): White noise series for *n*-th trial
1: /* Initialize IMF storage and parameters */
2: *IMF_list_*←∅, *n*←1
3: Normalize *x*(*t*) to zero mean
$4:\;\mathrm{WHILE}\;(n\leq \;N)\;\mathrm{DO}\;5:\;/*\;\mathrm{Add}\;\mathrm{controlled}\;\mathrm{noise}\;*/\;6:\;x_{n}(t)\leftarrow x(t)\;+\;\epsilon \cdot w_{n}(t)\;7:\;/*\mathrm{EMD}\;\mathrm{Decomposition}\;*/\;8:\;r(t)\leftarrow x_{n}(t)/*\;\mathrm{Initialize}\;\mathrm{residue}\;*/\;9:k\leftarrow 1/*\;\mathrm{IMF}\;\mathrm{counter}\;*/\;10:\;\mathrm{WHILE}\;(k\leq {\mathrm{IMF}}_{\max}\;\mathrm{AND}\;r(t)\;\mathrm{not}\;\mathrm{monotonic})\;\mathrm{DO}\;11:\;\;h(t)\leftarrow r(t)/*\;\mathrm{Working}\;\mathrm{signal}\;*/\;12:\;/*\mathrm{Extract}\;k\text{-}\mathrm{th}\;\mathrm{IMF}\;*/\;13:\;\mathrm{REPEAT}\;14:\;\mathrm{Identify}\;\mathrm{all}\;\mathrm{local}\;\mathrm{extrema}\;\mathrm{of}\;h(t)\;15:\;\mathrm{Interpolate}\;\mathrm{upper}\;\mathrm{envelope}\;e_{up}(t)\;(\mathrm{cubic}\;\mathrm{spline})\;16:\;\mathrm{Interpolate}\;\mathrm{lower}\;\mathrm{envelope}\;e_{\mathrm{lo}}(t)\;(\mathrm{cubic}\;\mathrm{spline})\;17:\;m(t)\leftarrow \frac{e_{up}\left(t\right)+e_{lo}\left(t\right)}{2}/*\;\mathrm{Mean}\mathrm{envelope}\;*/$ 18: *h*(*t*)←*h*(*t*) − *m*(*t*)
19: UNTIL (SD < 0.3)/* Stopping criterion */
20: Store *IMF_kn_*←*h*(*t*)
21: *r*(*t*)←*r*(*t*) − *IMF_kn_*
22:  *k*←*k* + 1
23: END WHILE
24: Store residue *Rn*(*t*)←*r*(*t*)
25: /*Calculate the sample entropy or Shannon entropy for each IMF order*/
26: FOR each IMF index k DO
27: Compute SampEn(IMF_kn)
28: END FOR
29: *n←n + 1*
30: END WHILE
31: *n*←*n* + 1
32: END WHILE
$33:\;/*\;\mathrm{Ensemble}\;\mathrm{Averaging}*/\;34:\;\mathrm{FOR}\;\mathrm{each}\;\mathrm{IMF}\;\mathrm{index}\;k\;\mathrm{DO}\;35:\;{\overset{————}{IMF}}_{k}\left(t\right)\leftarrow \frac{1}{N}\sum_{n=1}^{N}IMF_{k}^{n}\left(t\right)$

$36:\;\mathrm{Append}\;{\overset{————}{IMF}}_{k}\left(t\right)$ to *IMF_list_*
37: END FOR 
$38:\;\bar{R}\left(t\right)$$\leftarrow \frac{1}{N}\sum_{n=1}^{N}R^{n}\left(t\right)$/* Final residue */
$39:\;\mathrm{RETURN}\;{\mathrm{IMF}}_{\mathrm{list}},\;\bar{R}\left(t\right)$

### 2.2. Online News Sentiment Mining Model Theory

**Figure 3:**
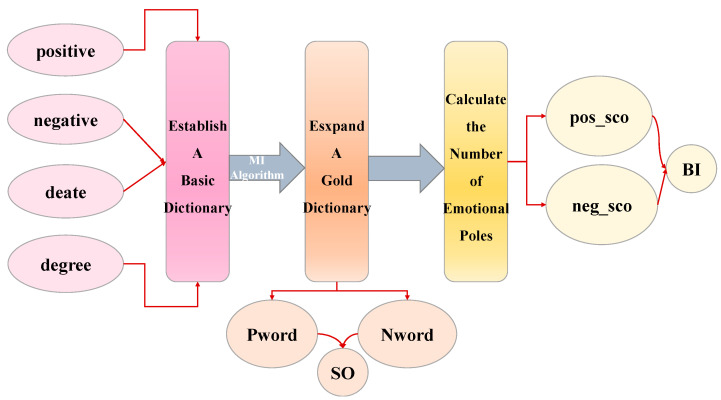
A technical process for calculating sentiment polarity based on online news sentiment mining models.

This module will detail how to calculate sentiment poles using an online news sentiment mining model *BI* [31]. The specific process is shown in Figure 3.

#### 2.2.1. Construction of a Basic Lexicon

##### Chinese Participle

In a sentiment polarity analysis based on online news texts, it is necessary to construct a multidimensional vector space representation on the basis of words to transform the textual data into computer-recognizable vectors for the next construction of the underlying lexicon. We chose to use the Tsinghua Finance and Economics Thesaurus as the base lexical entries [32]. Some of the terms are shown in Table 3.

##### Construction of an Emotional Lexicon

The Basic Emotion Dictionary includes the Basic Positive Emotion Dictionary and the Basic Negative Emotion Dictionary. We used the HowNet Sentiment Dictionary as our research dictionary. The Basic Positive Emotions Dictionary includes positive emotion words and positive evaluation words; the Basic Negative Affective Dictionary includes negative affective words and negative evaluative words. Some of the terms are shown in Table 4.

##### Construction of an Auxiliary Dictionary

The auxiliary dictionary constructed in this paper mainly consists of a Degate dictionary, a deactivation dictionary, and a degree-level dictionary. Some of the terms are shown in Table 4.

#### 2.2.2. Theory of MI Algorithms: An Information-Theoretic Foundation

Mutual Information (MI) is a fundamental concept in information theory, derived from Shannon entropy. It quantifies the reduction in uncertainty about one random variable given knowledge of another. Formally, for two discrete variables *X* and *Y*, the MI $I\left(X;Y\right)$ is defined as the divergence between their joint distribution and the product of their marginal distributions:

(14) $$I\left(X;Y\right)=\sum_{y\in Y}\sum_{x\in X}p\left(x,y\right)\log\left(\frac{p(x,y)}{p(x)p(y)}\right),$$

A higher MI value indicates a stronger statistical dependency and greater reduction in uncertainty. In the context of sentiment lexicon expansion, we leverage MI to measure the association strength between candidate words and pre-defined sets of positive or negative seed words. Words that significantly reduce the uncertainty about sentiment polarity (i.e., have high MI with a seed set) are deemed semantically aligned and incorporated into the expanded financial sentiment dictionary.

The process of calculating the degree of association between two terms is as follows:

(15) $$MI\left(w1,w2\right)=\log_{2}(\frac{p(w1,w2)}{p(w2)p(w2)}),$$

where *w*1 and *w*2 denote two different words. *p*(*w*1) and *p*(*w*2) denote the probabilities of *w*1 and *w*2 appearing in the news text.

(16) $$p(wi)=\frac{N_{wi}}{N_{total}},i=1,2,$$

where $N_{wi}$ indicates the number of news texts appearing in *wi*, $N_{total}$ indicates the total number of news texts. *p*(*w*1,*w*2) denotes the probability that *w*1 and *w*2 appear together in a news text, referred to as their co-occurrence probability,

(17) $$p(w1,w2)=\frac{N_{w1,w2}}{N_{total}},$$

where $N_{w1,w2}$ represents the number of news texts where *w*1 and *w*2 appear simultaneously.

By calculating the MI value of an unknown word with respect to the set of positive and negative sentiment words, the semantic disposition of that unknown word can be obtained SO,

(18) $$SO(w)=\frac{\sum_{pw\in Pw}MI(w,pw)}{N_{pw}}+\frac{\sum_{nw\in Nw}MI(w,nw)}{N_{nw}},$$

where *Pw* is positive seed thesaurus, *pw* is positive seed words concentrated in positive thesaurus; *Nw* is negative seed thesaurus, *nw* is negative seed words concentrated in negative thesaurus. $\sum_{pw\in Pw}MI(w,pw)$ is the sum of the MI values for each *pw* in *w* and *Pw*, $\sum_{nw\in Nw}MI(w,nw)$ is the sum of the MI values for each *nw* in *w* and *Nw*. $N_{pw}$ and $N_{nw}$ are the number of positive and negative seed words.

SO(w)>0 indicates that the MI between the unknown word and the positive seed word set is higher, thus classifying the unknown word as a positive term. SO(w)<0 indicates that the MI between the unknown word and the negative seed word set is higher, thus classifying the unknown word as a negative term.

#### 2.2.3. Calculation of the Number of Gold News Sentiment Poles

**Figure 4:**
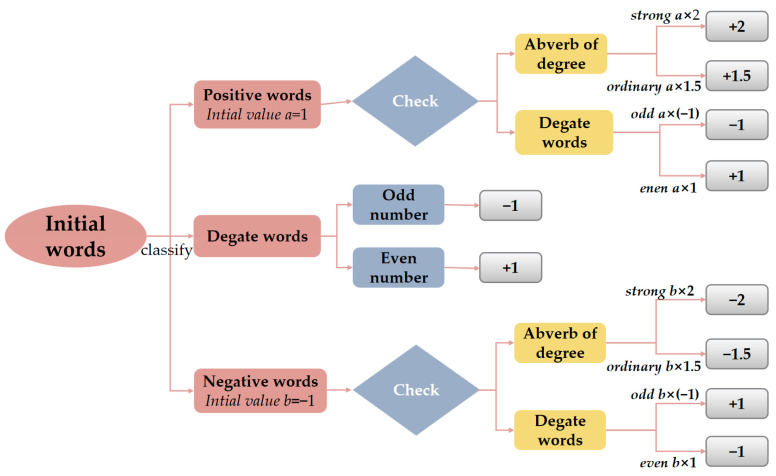
Calculation process for the emotional polarity *BI*.

To get the daily sentiment polarity, you need to calculate the sentiment polarity of each gold news clause, linearly sum to get the sentiment base of each news, and finally add the sentiment polarity of daily gold news to get the daily gold news sentiment polarity. The computational flow is shown in Figure 4.

Based on the above process, the sentiment scores of the sentences are obtained, and the sentence scores of each news item are summed to obtain the news sentiment score. Set when Score>0.5 is positive news and when Score<−0.5 is negative news. Thus, we can calculate the daily count of positive news items $N_{pos}$ and negative news items $N_{neg}$. The daily emotional polarity *BI* is

(19) $$BI=\ln\left(\frac{N_{pos}+0.5}{N_{neg}+0.5}\right),$$

when there is a lot of positive news released in the market BI>0, on the other hand, BI<0. Algorithm 2 presents the pseudocode for the Online News Sentiment Mining Model.
**Algorithm 2.** Online News Sentiment Mining Model pseudo-code.Online News Sentiment Mining ModelParameters:*L*: Maximum sequence length*E*: Word embedding dimension*K*: Number of sentiment classes (e.g., 3 for [negative, neutral, positive])*α*: Learning rate
Input:
Raw news text *D* = {*d*1,…,*dN*}Corresponding labels *Y* = {*y*1,…,*yN*} where yi∈{0,1,2}* yi*∈{0,1,2}
1:/*Text Preprocessing*/ 2: FOR each document *di*∈*D* DO
3: Remove non-alphabetic characters
4: Convert to lowercase
5: Tokenize using NLTK/Spacy →*tokens* = [*w*_1_,…,*w_m_*]
6: Lemmatize tokens
7: Remove stopwords
8: Pad/truncate to length *L*
9: END FOR
10:/*Feature Extraction*/ 11: Initialize pretrained embedding matrix *W*∈ℝ^∣*V*∣ ×*E*^ (GloVe/BERT)
12: FOR each token sequence *tokens_i_* DO
13: Xi←LookupEmbedding(W,tokens_i_)*Xi*←*LookupEmbedding*(*W*,*tokens_i_*)/* Shape: (L, E) */
14: END FOR
15:/*Hybrid Neural Network*/
16: Define model architecture: 17: Input layer: *input*←(*None*,*L*,*E*) 18:/* Parallel Feature Extractors */ 19: CNN Branch: 20: *conv*1←*Conv*1*D*(*filters* = 128,*kernel*_*size* = 3,*activation* = ‘*relu*’)(*input*) 21: *pool*1←*MaxPooling*1*D*(*pool*_*size* = 2)(*conv*1) 22: *drop*1←*Dropout*(0.5)(*pool*1) 23: LSTM Branch: 24: *lstm*1←*Bidirectional*(*LSTM*(*units* = 64,*return*_*sequences* = *True*))(*input*) 25: *att*←*AttentionLayer*()(*lstm*1)/* Self-attention mechanism */ 26:/* Feature Fusion */ 27: *merged*←*Concatenate*()([*drop*1,*att*]) 28: *dense*1←*Dense*(128,*activation* = ‘*relu*’)(*merged*) 29: *output*←*Dense*(*K*,*activation* = ‘*softmax*’)(*dense*1) 30:/*Model Training/* 31: Compile with: 32: *loss*←‘*categorical*_*crossentropy*’ 33: *optimizer*←*Adam*(*α*) 34: *metrics*←[‘*accuracy*’,*F*1_*score*] 35: Train using mini-batches: 36: FOR *epoch* = 1 TO *max*_*epochs* DO 37: Xbatch,Ybatch←SampleBatch(X,Y,batch_size = 32)*Xbatch*,*Ybatch*←*SampleBatch*(*X*,*Y*,*batch*_*size* = 32) 38:  *grads*←*Backpropagate*(*loss*,*output*) 39:  *UpdateWeights*(*optimizer*,*grads*) 40: IF *EarlyStopping*(*val*_*loss*,*patience* = 3) THEN BREAK 41: END FOR

### 2.3. CNN-QRLSTM Modeling Theory

Convolutional long short-term neural network based on quantile regression (CNN-QRLSTM) is a kind of deep learning model used to deal with time series data prediction. The model consists of a Convolutional Neural Network (CNN) and a Quartile Regression Model for Long- and Short-Term Memory Networks (QRLSTM).

#### 2.3.1. Convolutional Neural Network (CNN)

CNN is used for feature extraction and dimensionality reduction of time series data; it can recognize patterns on different time scales and is suitable for capturing local features of time series data. The main components of a CNN model include an input layer, a convolutional layer, a pooling layer, a fully connected layer, and an output layer. The calculation process is as follows:

First use a one-dimensional convolutional operation (Conv1D), for the input sequence $x\in R^{T\times d}$, convolution kernel $W\in R^{k\times d\times m}$ outputs,

(20) $$y_{t}^{j}=\sum_{i=0}^{k-1}\sum_{l=1}^{d}W_{i,l}^{j}\cdot x_{t+i,l}+b^{j},$$

where *T* is sequence length, *d* is feature dimension, *k* is the size of the nucleus, *m* is the number of output channels, $y_{t}^{j}$ denotes the output of the jth channel at time step t, $b^{j}$ is bias entry. If stride = 1, padding = 0, output length $T^{’}=T-k+1$.

Moving on to the pooling layer, reducing sequence length and increasing robustness using one-dimensional maximal pooling (ManPool1D).

(21) $$y_{t}=\max\left(x_{t\cdot s},x_{t\cdot s+1},\ldots ,x_{t\cdot s+k-1}\right),$$

where *s* is the step size and *k* is the pooling kernel size.

The final mapping to the prediction target is done through the full connectivity layer,

(22) $$\hat{y}=W_{fc}\cdot Flatten(y)+b_{fc}$$

#### 2.3.2. Quartile Regression of Long- and Short-Term Memory Networks (QRLSTM)

LSTM is used to capture long-term dependencies in time-series data, which regulates the flow of information through three gating units, i.e., the forgetting gate, input gate, and output gate, and a cellular state, to effectively deal with the problem of information transfer and memorization of time-series data. The calculation process is as follows [33]:

The forget gate decides what information to discard from the cell state,

(23) $$f_{t}=\sigma \left(W_{f}\cdot \left[h_{t-1},x_{t}\right]+b_{f}\right),$$

where *W_f_* is oblivion gate weighting matrix, *h_t_*_−1_ is the hidden state of the previous time step, *X_t_* is current input value.

Updating new information on cell status via Input Gate,

(24) $$i_{t}=\sigma \left(W_{i}\cdot \left[h_{t-1},x_{t}\right]+b_{i}\right)\;(\mathrm{Update}\;\mathrm{ratio}),$$

(25) $${\tilde{C}}_{t}=\tanh\left(W_{C}\cdot \left[h_{t-1},x_{t}\right]+b_{C}\right)\;(\mathrm{Candidate}\;\mathrm{value}),$$

Combining forgetting and input gates to update memory for cell state updating,

(26) $$C_{t}=f_{t}⊙C_{t-1}+i_{t}⊙{\tilde{C}}_{t},$$

where ⊙ is element-by-element multiplication.

Output hidden state based on cell state, i.e., Output Gate,

(27) $$O_{t}=\sigma \left(W_{o}\cdot \left[h_{t-1},x_{t}\right]+b_{o}\right),$$

(28) $$h_{t}=o_{t}⊙\tanh\left(C_{t}\right),$$

In this paper, we construct a QRLSTM model based on LSTM combined with quantile regression, estimating model parameters by optimizing the objective function,

(29) $$\left\{\begin{array}{l} F=\underset{W,b}{\min\;}E_{\lambda }\left(W,b\right)\\ E_{\lambda }\left(W,b\right)=\sum_{i=1}^{N}\rho_{\tau }\left[Y_{i}-f\left(X_{i},W,b\right)\right]\\ \rho_{\tau }\left(u\right)=u\left[\tau -I\left(u\right)\right] \end{array}\right.,$$

where $E_{\lambda }(W,b)$ is error function, $Y_{i}$ is sample actual values, $f\left(X_{i},W,b\right)$ is the output values of the LSTM model, *W* is weighting matrix, *b* is model bias term, *I*(*u*) is characteristic function, τ is the quantile of (0,1).

Get the conditional quantile of Y as an input function for kernel density estimation,

(30) $$\left\{\begin{array}{l} {\hat{Q}}_{Y}\left(\tau |X\right)=f\left(X,\hat{W}\left(\tau \right),\hat{b}\left(\tau \right)\right)\\ Z_{t}={\hat{Q}}_{Y}\left(\tau |X\right),t=1,2,\ldots ,T \end{array}\right.,$$

Assuming $\left(Z_{1},Z_{2},\ldots ,Z_{T}\right)$ is mutually independent, its kernel density estimate function is

(31) $${\hat{f}}_{h}\left(Z\right)=\frac{1}{Th}\sum_{t=1}^{T}K\left(\frac{Z-Z_{t}}{h}\right),$$

where *h* is wide, based on experience this paper selects $h=1.06\hat{\sigma }n^{-\;\frac{1}{5}}$. $\hat{\sigma }$ is sample variance, *n* is sample size, *T* is the number of quartiles, $K\left(\cdot \right)$ is nonnegative kernel function. In this paper, the Epanechnikov kernel function was selected to calculate the

(32) $$K\left(u\right)=\frac{3}{4}(1-u^{2})I\left(\left|u\right|\leq 1\right),$$

In this paper, we use the pinball loss to evaluate the probabilistic prediction effect, i.e., the smaller the loss, the better the effect. For the loss function (Pinball Loss) defined as

(33) $$L_{\tau ,t}\left(y_{t},{\hat{y}}_{t}\right)=\left\{\begin{array}{l} \tau \cdot \left|y_{t}-{\hat{y}}_{t}\right|\\ (1-\tau )\cdot \left|y_{t}-{\hat{y}}_{t}\right| \end{array}\right.\begin{array}{c} \;\;\;if\;y_{t}\geq {\hat{y}}_{t}\\ \;\;\;if\;y_{t}<{\hat{y}}_{t} \end{array},$$

(34) $$L=\frac{1}{QS}\sum_{\tau =1}^{Q}\sum_{t\in S}L_{\tau }\left(y_{t},{\hat{y}}_{t}\right),$$

where *y* is real value, $\hat{y}$ is predicted quartile, *Q* is target quartile values, *S* is the length of the test set.

#### 2.3.3. Entropy as a Measure of Predictive Uncertainty

While the quantile regression mechanism provides a range of possible outcomes, it is equally important to quantify the inherent uncertainty or informational clarity of these probabilistic predictions. To this end, we introduce information entropy as a complementary metric to evaluate the uncertainty encapsulated in the model’s output distribution.

For each prediction time step *t*, the CNN-QRLSTM model generates a set of quantile estimates $\left\{{\hat{Q}}_{\tau }\left(t\right)\right\}$ for τ∈T. These quantiles implicitly define an empirical cumulative distribution function (CDF). We can approximate the corresponding probability density and compute the conditional entropy of the prediction, given the model and input data,

(35) $$H\left(\hat{Y_{t}|X_{t}}\right)=-\sum_{k}\hat{p_{t,k}}\log_{2}\left(\hat{p_{t,k}}\right)$$

where $\hat{p_{t,k}}$ represents the probability mass assigned to the k-th bin of a discretized support derived from the predicted quantiles. A lower conditional entropy $H\left(\hat{Y_{t}|X_{t}}\right)$ indicates that the model’s predictive distribution is more concentrated, whereas a higher entropy reflects greater dispersion and uncertainty.

This entropy-based perspective aligns with and enriches the quantile regression framework, as follows:

(1) Complement to Pinball Loss: While the pinball loss optimizes for calibration at specific quantiles, minimizing the conditional entropy encourages the overall prediction distribution to be sharper and more informative, without sacrificing calibration.

(2) Uncertainty Decomposition: The total uncertainty (differential entropy) in the gold price series can be conceptually decomposed into aleatoric uncertainty (inherent noise in the data, captured by the spread of predictive intervals) and epistemic uncertainty (model uncertainty, potentially reduced with more data or a better model). The entropy of our quantile-based distribution primarily reflects the aleatoric component pertinent to risk assessment.

(3) Information Gain Interpretation: The reduction in entropy from the marginal distribution of gold prices *H*(*Y*) to the conditional predictive distribution *H*(*Y|X*) can be viewed as the information gain achieved by our model, quantifying how much uncertainty is resolved by incorporating the multi-source explanatory variables and temporal features.

**Figure 5:**
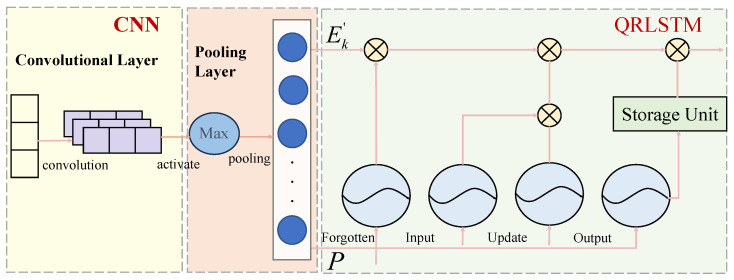
The total workflow of this module.

Thus, by integrating entropy analysis, our CNN-QRLSTM framework not only provides interval forecasts but also offers an information-theoretic gauge of prediction confidence. This empowers investors to distinguish between periods where the model makes precise, low-entropy forecasts and periods of high uncertainty, enabling more nuanced risk-aware decision-making. The complete CNN-QRLSTM workflow is shown in Figure 5, and the model pseudocode is presented in Algorithm 3.
**Algorithm 3.** The pseudo-code of CNN-QRLSTM.
CNN-QRLSTM for Multi-Quantile Time Series ForecastingParameters:*δ*—Convergence tolerance for early stopping*K*—Number of target quantiles (e.g., [0.05, 0.25, 0.5, 0.75, 0.95])*w*—Window size (lookback period)*f*—Number of input features
Hyperparameters:
*nepochs*—Maximum training epochs*batch*_*size*—Mini-batch size*η*—Learning rate1: /* Initialize model and training parameters */ 2: Set *epoch*←0, *converged*←*False* 3: Initialize network weights *θ* randomly 4: Normalize input data *X*∈ℝ*^N^*^× *w* × *f*^ and targets *y* $5:\;\mathrm{WHILE}\;(\mathrm{epoch}<\max\_\mathrm{epochs}\;\mathrm{AND}\;\mathrm{NOT}\;\mathrm{converged})\;\mathrm{DO}\;6:\;/*\;\mathrm{Mini}\text{-}\mathrm{batch}\;\mathrm{processing}\;*/\;7:\;\mathrm{FOR}\;\mathrm{each}\;\mathrm{batch}\;\;X_{b}\in {\mathbb{R}}^{\mathrm{batch}\_\mathrm{size}\;\times \;w\;\times \;f}\;\mathrm{DO}\;8:\;/*\;\mathrm{Forward}\;\mathrm{Pass}\;*/\;9:\;/*\;\mathrm{CNN}\;\mathrm{Feature}\;\mathrm{Extraction}\;*/\;10:\;\;C_{1}\;=\;\mathrm{ReLU}(\mathrm{Conv}1D_{128}(X_{b}))\;11:\;C_{2}\;=\;\mathrm{Dropout}(\mathrm{MaxPool}1D(\mathrm{ReLU}(\mathrm{Conv}1D_{64}(C_{1}))))\;12:\;/*\;\mathrm{LSTM}\;\mathrm{Sequence}\;\mathrm{Processing}\;*/\;13:\;\;L_{1}\;=\;{\mathrm{LSTM}}_{256}(\mathrm{Reshape}(C_{2}))\;14:\;\;H\;=\mathrm{Dropout}({\mathrm{LSTM}}_{128}(L_{1}))\;15:\;/*\;\mathrm{Multi}\text{-}\mathrm{Quantile}\;\mathrm{Output}\;*/\;16:\;\;{\hat{y}}^{k}\;=\;\mathrm{Dense}(H)\;\mathrm{for}\;\mathrm{each}\;k\in K$

$17:\;/*\;\mathrm{Loss}\;\mathrm{Computation}\;*/\;18:\;\mathrm{FOR}\;\mathrm{each}\;\mathrm{quantile}\;\;k\in K\;\mathrm{DO}\;19:{ℒ}^{k}\;=\;\frac{1}{\left|batch\right|}\sum \max[k(y\;-\;{\hat{y}}^{k}),(k\;-\;1)(y\;-\;{\hat{y}}^{k})]$ 20: END FOR 21: *total*_*loss* = ∑*_k_*_∈*K*_ℒ*^k^* 22: /* Backpropagation */ 23: Compute gradients ∇*_θ_total*_*loss* 24: Update parameters *θ*←*θ* − *η*∇*_θ_* 25: END FOR 26: /*Epoch Evaluation*/ 27: IF (∣*total*_*loss_epoch_* − *total*_*loss_epoch_* _− 1_∣ < *δ*) THEN 28:  *converged*←*True* 29: END IF 30: *epoch*←*epoch* + 1 31: END WHILE $32:\;/*\;\mathrm{Model}\;\mathrm{Output}\;*/\;33:\;\mathrm{Return}\;\mathrm{trained}\;\mathrm{weights}\;\theta \;\mathrm{and}\;\mathrm{quantile}\;\mathrm{predictions}\;\{{\hat{y}}^{k}\}k\in K$

#### 2.3.4. CNN-QRLSTM Collaborative Mechanism and Performance Enhancement Approaches

CNN, QR, and LSTM synergistically enhance prediction performance within this framework through the following mechanisms.

(1) CNN as a Local Semantic and Temporal Pattern Extractor: Perform one-dimensional convolution on input sequences (including sentiment indicators, etc.) to capture short-term volatility patterns and local dependencies. Through dimension reduction via the pooling layer, salient features are preserved while noise transmission to subsequent LSTM layers is minimized.

(2) LSTM as a module for long-term dependencies and sequence memory: Accepting high-dimensional features extracted by CNN, it learns the long-term dynamic relationships between gold prices and multiple factors. Regulate information flow through gating mechanisms to prevent gradient vanishing and enhance modeling capabilities for complex sequence structures.

(3) QR as the output layer for uncertainty quantification and risk perception: Based on the LSTM hidden state, multiple conditional quantiles are output through a quantile fully connected layer. Using the Pinball loss function to jointly optimize all quantiles enables the model to not only predict trends but also assess price ranges at different confidence levels.

CNN’s local perception capability enhances the LSTM’s sensitivity to short-term fluctuations, while the QR mechanism transforms the LSTM’s hidden state into probabilistic outputs. This adds risk dimension information to point forecasts, thereby enhancing decision support value.

### 2.4. Framework of the Proposed Model

**Figure 6:**
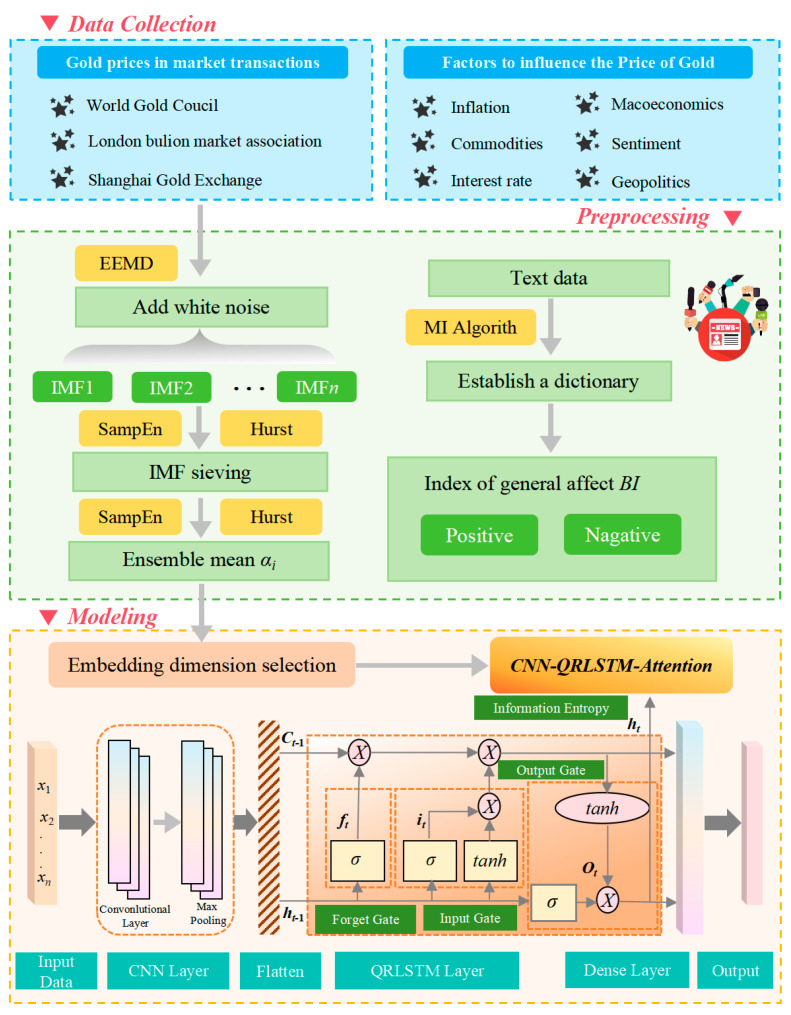
Technical flow of this research.

In this paper, we propose an integrated prediction framework based on the CNN-QRLSTM model and the online news sentiment mining model. The framework is organized into three modules. Firstly, for the data collection module, we selected three major gold exchanges, namely the World Exchange, the London Bullion Exchange, and the Shanghai Exchange. In order to provide the accuracy of the model’s predictions, we included the factors influencing the price of gold in the parameter indicators, choosing a total of eight indicators in the categories of inflation, macroeconomic categories, commodities, media sentiment, interest rates, and economic policy. Secondly, in the data processing module, for the numerical data, we use the EEMD method to add white noise to the original data, perform multiple repetitions of decomposition into multiple sets of IMFs, and introduce the Hurst index, and the IMFs with the highest frequency and the lowest Hurst index are rejected as white noise, and then the reconstructed data are used as the input data for the prediction model. To improve the accuracy of the predictive model, we will calculate the Hurst index of the processed dataset to determine the embedding dimension of the model. For the textual data, we build a textual dictionary of gold prices by the MI algorithm and calculate the sentiment polarity BI to be included in the independent variables of the prediction model. Thirdly, in the machine learning module, we use the CNN-QRLSTM model for gold price prediction, and experiments prove that this framework improves the accuracy and stability of the prediction model, which is better than the traditional machine model. A full-text technical flowchart is shown in Figure 6.

## 3. Results

### 3.1. Data Selection and Model Parameters

#### 3.1.1. Gold Price Date

**Figure 7:**
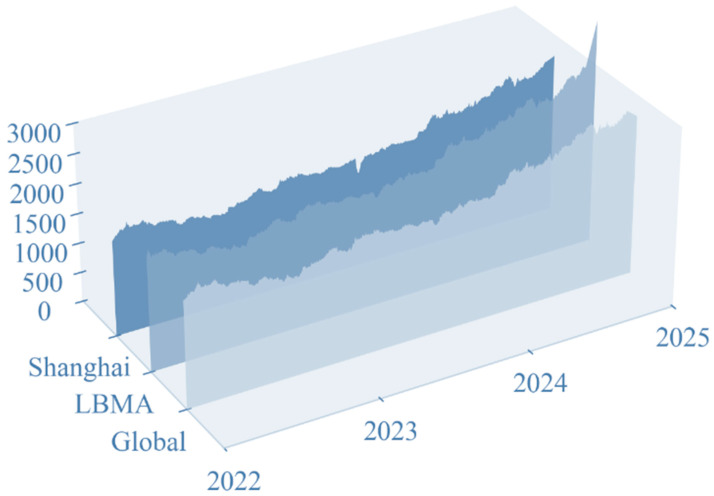
WGC, LBMA and SGE Gold Price Trends from 1 February 2022, to 28 February 2025.

In order to prove the accuracy of the prediction model we constructed and to realize a reasonable prediction of the gold price, we chose the world gold market and two gold markets as the object of study. The dataset includes daily traded gold prices from 1 February 2022, to 28 February 2025, for a total of 1093 days (including non-gold trading days). Figure 7 provides information on time horizon changes for three markets: the WGC, the London Bullion Association Market (LBMA), and the Shanghai Gold Exchange (SGE).

Our research centers around short-term gold forecasts, particularly for day-ahead price forecasts. A total of 70% of the dataset was used for model construction and 30% for evaluating the performance of the predictive model. Due to the existence of the gold market closure and different countries’ gold market closure times, there are a large number of missing values in each dataset. In order to ensure that the data of each market can be fairly compared horizontally, we use Lagrange interpolation to fill in the missing values, selecting the missing values before and after the five data points as a reference to ensure that the filled value is in line with the market’s gold price volatility.

Through a series of data processing, we not only improve the quality of the gold market price but also provide a solid foundation for subsequent research on the accuracy of gold price forecasting and the exploration of the influence of various factors on the volatility of the gold price.

#### 3.1.2. Data on Impact Factors

**Figure 8:**
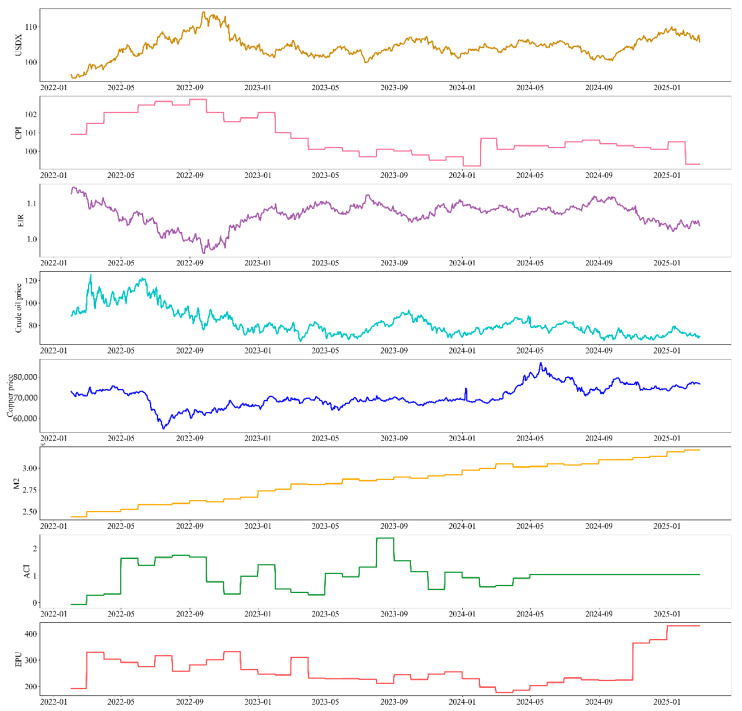
Trends in Impact Factors 1 February 2022 through 28 February 2025.

Also included within the dataset are eight influencing factors related to gold price volatility. We have taken into account the macroeconomic type of factors that affect the gold price (dollar index, inflation, etc.), economic and political factors, and some other related factors (commodities, media sentiment, etc.). Due to the existence of market closures for some commodities, the data is the same as the gold price using Lagrange interpolation to supplement missing values. Most of these factors are positively or negatively correlated with gold, and taking them into account in the model can improve the interpretability of the predictive model. The following is a detailed description of these influencing factors in Figure 8.

● **US dollar index (USDX)**: Gold is a commodity denominated in U.S. dollars, and the U.S. dollar index is a measure of the U.S. dollar in the international foreign exchange market. Exchange rate changes in a comprehensive indicator, so the U.S. dollar index is one of the factors closely related to the price of gold. A stronger U.S. dollar index means that the U.S. dollar strengthens relative to other major currencies, making it more expensive to buy gold, dampening demand for gold, which tends to fall in price. Therefore, USDX has a significant negative correlation with the price of gold, and the inclusion of the U.S. dollar index in the model in this study helps to improve the accuracy of the model’s predictions.
● **Inflation (CPI)**: Gold is seen as an effective tool in the fight against inflation. When CPI figures rise, inflationary pressures increase, and investors tend to shift their assets to value-protecting commodities such as gold, which raises the demand for gold, and the price of gold tends to rise. Thus the CPI is significantly and positively correlated with the price of gold in the short run.
● **Effective interest rate (EIR)**: “Real interest rate” refers to the real interest rate at which investors receive interest returns after excluding inflation, and the level of EIR directly affects investors’ investment decisions. When real interest rates rise, investors are more inclined to deposit their money in banks or more profitable financial products, the demand for gold falls, and the price falls; instead, investors will shift their money to gold based on avoidance. Thus, EIR is negatively correlated with the price of gold.
● **Monetary policy (M2)**: The easing or tightening of monetary policy directly affects the country’s money supply. China’s current monetary statistics system divides the money supply into three levels, M0, M1, and M2, of which the growth rate of M2 is often used to measure the degree of monetary policy easing. When M2 grows rapidly, it indicates a looser monetary policy, more money on the market, and lower interest rates when investors seek more value-preserving assets, and the price of gold rises. Therefore, M2 is positively correlated with the price of gold, and the looser the monetary policy, the faster the price of gold grows.
● **Bulk commodities (Petroleum and Copper)**: Commodity markets are closely linked to global economic conditions and the monetary environment. Prices of energy commodities (e.g., oil) and metal commodities (e.g., copper, aluminum) are typically influenced by global economic growth and industrial production activity. When the global economy booms, industrial production increases, and the demand for energy and metal grows; price increases at the same time will also trigger the rise of inflation. Then investors usually choose to preserve value, gold demand increases, and the price rises. Whereas agricultural commodities have a relatively weak relationship with the price of gold, in the case of an extreme food crisis, which could lead to rising prices and severe inflation, investors would tend to acquire gold, leading to an increase in the price of gold. Thus, in general, commodities are positively correlated with the price of gold, with petroleum as a proxy for energy commodities and copper as a proxy for metals in our dataset, leaving out for the time being agricultural commodities, where the correlation is weak.
● **Economic policy (EPU)**: The Economic Policy Uncertainty Index (EPU) reflects global economic and political events such as trade disputes and political unrest. Rising EPU means increased economic policy uncertainty and increased uncertainty about the future economic outlook, which prompts investors to move their money to relatively value-protecting gold, and the price of gold thus rises. Since the implementation of economic policies does not happen overnight and requires a long-term process, EPU and gold prices show a positive correlation in the long run, but the relationship is not significant in the short run.
● **Climate risk (ACI)**: Extreme weather events caused by climate change, such as high temperatures and heavy rains, have had a significant impact on the gold mining industry. Extreme weather events often increase the difficulty of miners’ work as well as their equipment requirements, increase economic costs, and can even result in economic losses when the price of gold rises as a result of increased mining costs. In addition to this, investors will invest in gold as a safe-haven asset in the face of uncertain weather events, and the price of gold will rise. We use the Actuarial Climate Index (ACI) as a numerical indicator of climate risk factors, which looks at six extreme climate events: extreme low temperatures (LT), extreme high temperatures (HT), extreme rainfall (Hr), extreme drought (Dr), strong winds (Hw), and sea level (Sl). We therefore believe that the ACI is positively correlated with the price of gold.
● **Media sentiment (BI)**: Media sentiment has a significant impact on the volatility of the gold price. The price of gold falls when there is a large amount of positive news about gold in the media, causing positive sentiment to permeate the market, which in turn leads investors to invest in high-risk, high-return assets such as equities and emerging industries when there is less demand for gold; conversely, when the media reports negative news in a big way, the price of gold rises. We built an online news sentiment model for calculating the sentiment polarity (BI) of text data and chose BI as a digital indicator reflecting media sentiment, with higher BI values indicating more negative media sentiment and more disturbed market sentiment. Therefore, BI is considered to be negatively correlated with the price of gold. We incorporate the calculation of daily BI values into the prediction framework using the method of crawling gold news texts.

Table 5 presents the factors influencing gold prices introduced in this model, categorizes them, and provides the digital indicators incorporated into the model.

#### 3.1.3. Risk Prevention and Control for Aligning Sentiment Data with Price Sequences

When constructing daily sentiment indicators *BI* and aligning them with gold price sequences, risks such as overfitting, information leakage, sentiment lag, and noise interference may arise.

If an emotion dictionary relies excessively on training set news, it may lead to reduced out-of-sample generalization capabilities and result in overfitting. This paper employs a rolling time window to partition the training and validation sets, ensuring the emotion dictionary undergoes continuous validation within the dynamic news corpus.

Using future news sentiment to predict current prices, or incorporating future price information into sentiment indicators, will distort prediction results and heighten the risk of information leakage. This study strictly employs time-series alignment to ensure that sentiment metrics for day *t* are calculated solely based on news released on that day and prior days. During model training, sentiment data from the preceding period is used as input to prevent forward-looking bias.

Due to the time lag between news releases and market reactions, irrational sentiment noise may interfere with model learning. This paper employs the EEMD–Hurst–Entropy method to denoise emotional sequences. By incorporating an attention mechanism into the CNN-QRLSTM architecture, it assigns dynamic weights to emotional features across different time steps, thereby enhancing the model’s ability to identify critical emotional signals.

### 3.2. Data Preprocessing Based on EEDM and Hurst Indexes

We first use the EEMD method to decompose the digital dataset consisting of gold prices and eight influencing factors in three gold markets, and then calculate the frequency and Hurst index of each IMF, and obtain the denoised digital dataset by controlling the Hurst index of the integrated dataset and removing the IMFs with high frequency and low Hurst index as white noise. The processed digital dataset retains the trend of the original dataset and has removed the most unstable fluctuation values, presenting a clearer and more stable trend, which helps to improve the accuracy and stability of the subsequent model predictions.

In the data preprocessing module, we first decompose the three market gold price datasets using EEMD, and then remove some of the IMFs as white noise in combination with the Hurst index to obtain a denoised dataset.

**Figure 9:**
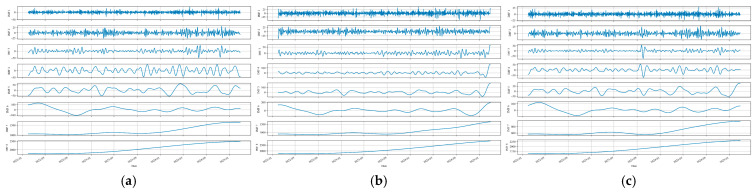
Decomposed IMF results. (**a**) Decomposition results for WGC data; (**b**) decomposition results for LBMA data; (**c**) decomposition results for SGE data.

We categorized the dataset into 8 groups IMF. Shows IMF results after decomposition of data for three markets in Figure 9. It can be seen that IMF1 and IMF2 show violent fluctuations with large frequencies that may contain noise. IMF8, on the other hand, has trended flat and can be considered a trend term.

The decomposed Hurst index of the IMF is shown in Table 6. It can be seen that the values of IMF1 to IMF4 are all less than 0.5; in contrast, the values of IMF7 and IMF8 are all around 1 and have leveled off. By removing each set of IMFs separately and calculating the Hurst index of the integrated dataset after the removal, the value was controlled to be between 0.5 and 0.7. Finally, we find that the Hurst index after removing IMF1 and IMF2 is 0.51; therefore, these two sets of IMFs are removed as white noise.

**Figure 10:**
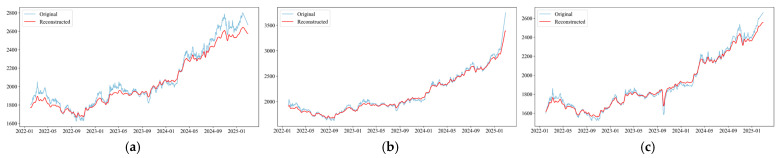
Comparison of data trends before and after EEMD-H model treatment. (**a**) Comparison of data before and after WGC processing; (**b**) comparison of data before and after LBMA processing; (**c**) comparison of data before and after SGE processing.

In Figure 10, comparing the original data with the data processed by the EEMD-H method, it can be observed that the processed data filters out the unstable components and outliers, presenting a clearer convergence result.

### 3.3. Calculation of Sentiment Poles Based on Online News Sentiment Mining Models

In the age of data, online news reports are the externalized manifestation of media sentiment, and different media reports investors tend to use online news reports as the main reference for investment, i.e., media sentiment indirectly influences investors’ investment decisions. Therefore, it is necessary to analyze gold news texts and incorporate the calculated sentiment polarity as a digital indicator of media sentiment into the prediction model.

**Figure 11:**
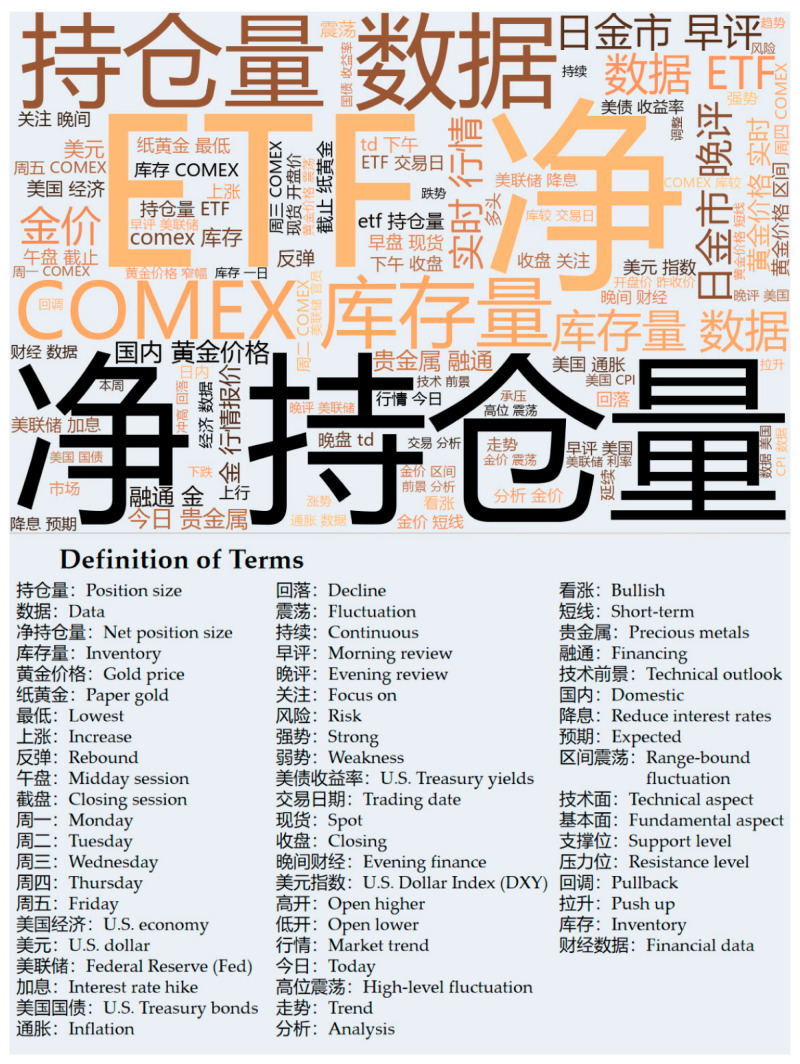
Online Newsword Cloud.

The textual data is difficult to crawl due to the anti-crawling techniques of foreign gold sites; we chose the Golden Investment Network as the corpus for our study. We collected 1303 news text data from the corpus, which contains the headlines as well as the body content of the news. Figure 11 displays a word cloud of crawled news entries.

Sentiment polarity analysis of the price of gold using the base dictionary is not accurate due to the limited number of words involved in the creation of the base dictionary, which is not domain-related. In order to better compute the sentiment polarity of gold investments, in this module we use the MI algorithm to expand the base lexicon into a gold price sentiment lexicon applicable to the gold domain.

The MI algorithm is a metric in information theory used to quantify the dependence between two random variables and is often used to compute the emotional tendency of Chinese words. It detects a nonlinear relationship between two words and measures the degree of association. We first utilized the statistics of sentiment words in 300 gold news articles to select the positive and negative sentiment words with prominent polarity as the seed word set, and some of the seed words are shown in Table 7.

At this point, we have obtained the golden sentiment lexicon through the MI algorithm, with a total of 55 positive sentiment words and 104 negative sentiment words, and some of the seed words are shown in Table 8. Combined with the base lexicon used in this paper, we finally constructed a sentiment lexicon for the gold domain, which serves as the basis for calculating the number of gold news sentiment poles.

**Figure 12:**
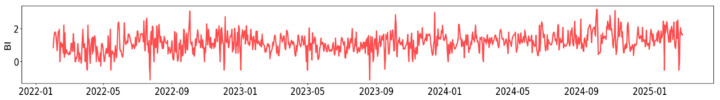
Daily *BI* calculations.

We include the calculated *BI* as a numerical indicator of media sentiment among the factors influencing the price of gold in our forecasting models. The daily *BI* trend results are shown in Figure 12.

### 3.4. Embedded Dimension Selection for Hurst Index Based Prediction Framework

Yang et al. (2023) demonstrated in their study on the relationship between the Hurst exponent and prediction accuracy that, when the Hurst index is lower than 1.92, the pattern of change within the data is more complex, so more embedding dimensions are needed to construct the prediction framework [34,35]; when the Hurst index is higher than 1.92, the pattern of change within the data is relatively clear, and therefore fewer embedding dimensions are needed to construct the prediction framework.

Since we control the Hurst index of the EEMD noise reduction processed data at 0.56, we choose to set the embedding dimension to 9 based on the correlation between the influences and the gold price.

**Figure 13:**
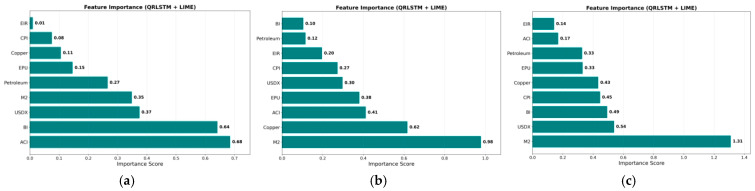
Correlation between various influencing factors and the price of gold. (**a**) WGC Correlation; (**b**) LBMA Correlation; (**c**) SGE Correlation.

From the Figure 13, it is evident that the USDX, interest rates, economic policies, and investor sentiment have relatively weak explanatory power for gold price fluctuations and exhibit a positive driving effect; monetary policy and copper prices have significant explanatory power for gold price fluctuations and present a positive driving effect; the Climate Risk Index (ACI) has relatively weak explanatory power for gold price fluctuations and shows a negative driving effect; inflation (CPI) and crude oil prices (Petroleum) have significant explanatory power for gold price fluctuations and demonstrate a negative driving effect.

### 3.5. Modeling Results Based on the Golden Sentiment Polarity BI

**Figure 14:**
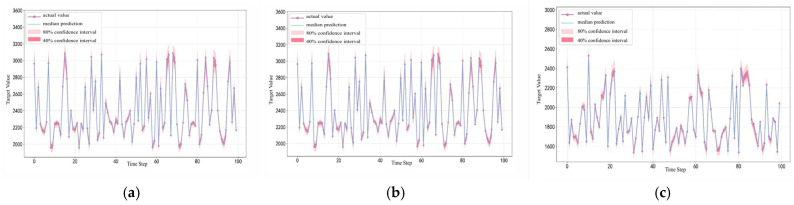
Point prediction and interval prediction results based on CNN-QRLSTM prediction models. (**a**) WGC Final Forecast Results; (**b**) LBMA Final Forecast Results; (**c**) SGE Final Forecast Results.

Figure 14 shows the point prediction and interval prediction results based on the CNN-QRLSTM prediction model, where we performed interval prediction at 80% and 40% confidence levels, respectively.

### 3.6. Numerical Verification

In this section, in order to validate the effectiveness of our proposed gold price prediction framework, we design evaluation experiments to compare and analyze the datasets. By observing the combined performance of the framework under different evaluation metrics, the EEMD, the necessity of introducing golden sentiment poles, and the superiority of the CNN-QRLSTM over the effect of a single model are identified.

The calculation process for all evaluation indicators (*MAE*, *MSE*, *RMES*, *MAPE*, *R*^2^) is as follows:

(36) $$MAE=\frac{1}{n}\sum_{i=1}^{n}\left|y_{i}-{\hat{y}}_{i}\right|,$$

(37) $$MSE=\frac{1}{n}{\sum_{i=1}^{n}\left(y_{i}-{\hat{y}}_{i}\right)}^{2},$$

(38) $$RMSE=\;\sqrt{\frac{1}{n}\sum_{i=1}^{n}{\left(y_{i}-{\hat{y}}_{i}\right)}^{2}},$$

(39) $$MAPE=\frac{100\%}{n}\sum_{i=1}^{n}\left|\frac{y_{i}-{\hat{y}}_{i}}{y_{i}}\right|,$$

(40) $$R^{2}=1-\frac{\sum_{i=1}^{n}{\left(y_{i}-{\hat{y}}_{i}\right)}^{2}}{\sum_{i=1}^{n}{\left(y_{i}-\bar{y}\right)}^{2}},$$

We control for the combination between EEMD and each single model, i.e., we compare the values of all evaluated metrics for prediction frameworks, namely, LSTM, CNN-LSTM, CNN-QRLSTM, EEMD-CNN-LSTM, EEMD-CNN-QRLSTM, XGBoost and ARIMA-GRACH. On this basis, the introduction of the golden sentiment polarity BI was controlled, and the specific experimental data are shown in Table 9, Table 10 and Table 11 (all interval predictions were taken with a confidence level of 50%).

Table 9, Table 10 and Table 11 list the experimental results of WGC, LBMA, and SGE gold price data in different forecasting models in various indicators, where the bolded values indicate that the model performance is optimal. Overall, the application of the complete model is a significant improvement over the original LSTM model. The MAE, MSE, RMSE, and MAPE show a decreasing trend, and the R^2^ shows an increasing trend. However, ARIMA-GARCH fails to capture the complex nonlinear correlations between exogenous features and target variables, while XGBoost overlooks the strong time-series lag dependence of the dataset and suffers from severe overfitting. In contrast, hybrid or deep learning models perform far better here, as they can well integrate time-series sequence characteristics and nonlinear feature interactions, achieving much lower error metrics and more robust predictive results.

Take the WGC market data as an example, as seen in Table 7. Comparing the single LSTM model without the introduction of gold sentiment poles and without data processing with the full forecasting framework BI-EEMD-CNN-QRLSTM, the MAE indicator decreases from 26.505 to 13.200, the MSE indicator decreases from 1225.715 to 281.660, the RMSE indicator decreases from 35.010 to 16.783, the MAPE indicator decreases from 1.254 to 0.542, and R^2^ increases from 0.989 to 0.998. This shows that our prediction framework significantly reduces errors and improves prediction accuracy overall.

In order to prove the necessity of the data processing module EEMD, we compare CNN-QRLSTM with EEMD-CNN-LSTM after the introduction of BI, and the results show that the MAE metric decreases from 32.200 to 13.200, the MSE metric will be 281.660 instead of 965.040, the RMSE metric will be 16.783 instead of 31.065, the MAPE metric from 1.201 to 0.542, and the R2 indicator from 0.991 to 0.998. From this we can see that after EEMD processing the data error is reduced and accuracy is increased, proving the necessity of EEMD processing.

In addition to this, to verify the performance improvement after adding quantile regression, we compare EEMD-CNN-LSTM with EEMD-CNN-QRLSTM after introducing BI. In order to verify the necessity of introducing golden sentiment poles, we compare EEMD-CNN-QRLSTM without BI with EEMD-CNN-QRLSTM with BI. This is all to show the results of decreasing MAE, MSE, RMSE, and MAPE and increasing R^2^. Thus, we further confirm the importance of quantile regression with BI.

Overall, the BI-EEMD-CNN-QRLSTM framework mostly outperforms the single model on all datasets and metrics, confirming the robustness and reliability of our proposed modeling framework for gold price prediction.

## 4. Discussion

In order to ensure the reliability and validity of the model’s prediction results, this section evaluates the model in several dimensions through prediction interval coverage tests, time dependence tests, etc. We will focus on the model’s goodness-of-fit and prediction accuracy to ensure its reliability in real gold price prediction applications.

### 4.1. Forecast Interval Coverage Test

This test is mainly used to accurately assess whether the prediction intervals output by the model (e.g., the corresponding interquartile ranges at different confidence levels) can realistically cover the range of fluctuations of the actual gold price. The effectiveness of the quantile regression model in predicting the range of price fluctuations is verified by comparing the proportion of actual prices falling within the predicted range with the theoretical confidence level to ensure that the model is able to effectively respond to large fluctuations in the market.

**Figure 15:**
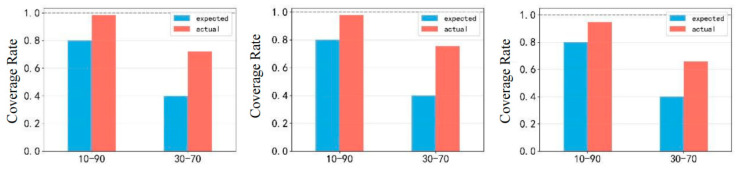
Coverage test.

In Figure 15, the model has a better prediction interval at the 30–70 quartile, while the interval at the 10–90 quartile is too wide and the prediction is relatively too conservative.

### 4.2. Time-Dependent Test

In order to test in depth whether there is temporal autocorrelation in the model residual series, so as to accurately determine whether the model has adequately extracted the long-run dependencies embedded in the time series, we conducted an ACF test.

**Figure 16:**
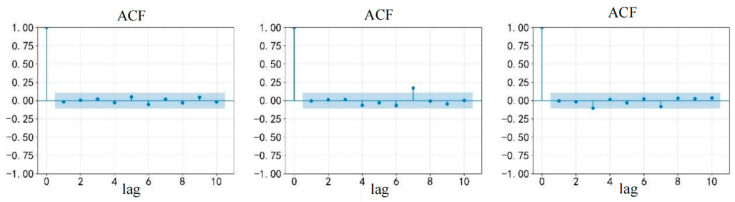
Residual autocorrelation coefficient ACF.

As shown in Figure 16, the residuals are close to 0 after the delay of the 6th and 12th order, indicating that the residuals are white noise (i.e., there is no significant autocorrelation), which fully indicates that the model has successfully captured the important temporal characteristics embedded in the data, effectively avoiding the omission of that key dynamic information affecting the price of gold, and guaranteeing the timeliness and accuracy of the model prediction.

### 4.3. Extreme Case Performance

To quantify the model’s ability to capture extreme market risks, Table 12 presents the performance of the EEMD-CNN-QRLSTM model under extreme scenarios (High-Volatility Rally, High-Volatility Drop) and a stable control scenario across the three major markets.

(1) High-Volatility Rally: An extreme upward scenario where the daily gold price increase exceeds 1.2 times the standard deviation of historical returns, reflecting sudden positive shocks such as geopolitical conflicts and favorable policies.

(2) High-Volatility Drop: An extreme downward scenario where the daily gold price decline exceeds 1.2 times the standard deviation of historical returns, corresponding to negative shocks such as better-than-expected economic data and liquidity tightening.

(3) Normal Stability (Control): A stable scenario where price fluctuations fall within ±0.5 times the standard deviation of historical means, serving as a benchmark for performance comparison.

The results show: (1) In extreme scenarios, the model still maintains excellent fitting performance with R^2^ all above 0.987, among which the R^2^ of the SGE market in the High-Volatility Drop scenario reaches 0.995, significantly outperforming the prediction performance of traditional models in extreme environments; (2) the error indicators exhibit a reasonable incremental trend of ‘Normal Stability < High-Volatility Rally < High-Volatility Drop’. For example, the RMSE of the LBMA market in the High-Volatility Drop scenario (47.060) increases by 77.2% compared with the stable scenario (26.560), which is consistent with the objective law of increased price fluctuation randomness under extreme events; (3) In cross-market comparison, the MAE of the SGE market in extreme scenarios (13.150–15.990) is significantly lower than that of WGC (17.160–20.580) and LBMA (27.970–32.390), reflecting the model’s stronger adaptability to extreme risks in China’s domestic gold market. The above results verify the core advantage of the QRLSTM quantile regression mechanism—by quantifying the conditional quantile distribution of prices, it effectively covers the range of extreme fluctuations, providing an accurate quantitative basis for risk hedging in extreme market environments.

### 4.4. Information-Theoretic Analysis of Uncertainty Reduction

We further analyze the model’s performance through an information-theoretic lens by computing the entropy reduction between the original gold price series and the model residuals. The entropy of the raw series *H*(*X*) represents the intrinsic market uncertainty. After processing with EEMD-Hurst and prediction with CNN-QRLSTM, the entropy of residuals H(ε) decreases significantly. The information gain IG=H(X)−H(ε) quantifies how much uncertainty the model explains. Our framework achieves an average IG of 1.8 bits across three markets, indicating substantial uncertainty resolution. This aligns with the MI-driven sentiment expansion, which adds informative features that reduce entropy in price prediction.

### 4.5. Computational Overhead and Real-Time Applicability of the CNN-QRLSTM Model

We quantitatively analyzed the computational overhead of the CNN-QRLSTM model under the identical hardware conditions (CPU: Intel i7-12700H, GPU: NVIDIA RTX 3060 6G, Memory: 16G) with a unified training setup (12,000 samples, batch size 32, 30 training epochs), compared its inference speed with lightweight baseline models, and further analyzed its applicability in real-time/high-frequency gold price forecasting scenarios, while proposing targeted lightweight optimization schemes and hardware recommendations.

In terms of parameter count, the single CNN and LSTM models have about 2.3 M and 5.8 M parameters, respectively, while the CNN-QRLSTM model reaches 8.6 M, increasing by 274% and 48.3%, respectively, due to the added custom attention layer, 5 quantile-corresponding fully connected layers in QRLSTM, and the CNN + LSTM double-layer structure. For training time, the single CNN takes 1.2 min, the single LSTM 3.5 min, and the CNN-QRLSTM 5.8 min (383% and 65.7% increases), caused by the complex model structure, more intricate pinball loss calculation, with the overall training time still within an acceptable range. In terms of inference overhead, the single-sample inference time of the single CNN, single LSTM and CNN-QRLSTM is about 12 μs, 28 μs and 45 μs (275% and 60.7% increases for the latter), and the 1000-sample inference time is about 8.5 ms, 22.3 ms and 36.8 ms (333% and 65% increases for the latter). The increased inference overhead stems from the longer forward propagation chain (CNN→QRLSTM), and the model shows a batch inference efficiency advantage with a significantly lower time growth rate in batch inference. We also compared the inference speed with lightweight machine learning models (XGBoost): the 1000-sample inference time of XGBoost is about 15.2 ms respectively, while that of CNN-QRLSTM is 36.8 ms. Though slower than lightweight models, it is significantly superior to other deep learning models such as Transformer-Time-Series (89.5 ms for 1000 samples), and its inference overhead is acceptable in terms of the cost-performance ratio of prediction information value to inference overhead, as it can output multi-quantile interval predictions while lightweight models only generate point predictions.

For real-time/high-frequency forecasting applicability, the CNN-QRLSTM model is well-suited for hour-level real-time forecasting in the gold market: the total processing time for a single sample is controlled within 100 μs even with data preprocessing and feature extraction, far lower than the hour-level forecasting time window; its batch inference efficiency is high and its structural design is adapted to the characteristics of high-frequency data. For minute-level ultra-high-frequency forecasting, lightweight optimization is required, including model pruning, parameter quantization, CNN structure simplification and lightweight attention layer replacement. After optimization, the model’s parameter count can be reduced to less than 5 M, the single-sample inference time within 20 μs, and the prediction accuracy loss limited to less than 5%, without affecting practical application value. We put forward hardware recommendations for different scenarios: conventional CPUs or entry-level GPUs are sufficient for hour-level real-time forecasting; GPU clusters or dedicated inference chips combined with lightweight optimization are recommended for minute-level ultra-high-frequency forecasting; mid-to-high-end GPUs are suggested for the model training phase to shorten training time and improve development efficiency.

## 5. Conclusions

This study proposes a hybrid forecasting framework that is grounded in information-theoretic principles and integrates deep learning to address the complexity and uncertainty inherent in gold price fluctuations. By explicitly quantifying and managing market entropy—a measure of informational randomness and unpredictability—our approach reframes gold price prediction as a problem of uncertainty reduction through information gain. The core innovation lies in the synergistic application of entropy-aware signal processing (EEMD-Hurst with Sample Entropy filtering), information-theoretic feature engineering (Mutual Information-based sentiment lexicon expansion), and entropy-interpretable probabilistic modeling (CNN-QRLSTM with quantile regression). This triad ensures that both structured numeric data and unstructured textual information are transformed into low-entropy, high-information features that maximally reduce predictive uncertainty.

At the data level, historical transaction data from the world’s three major gold markets were selected as the prediction targets. The model systematically incorporates multidimensional influencing factors such as the U.S. Dollar Index, inflation, commodity prices, and media sentiment to enhance its explanatory power in real-world scenarios. During the preprocessing stage, the original signal was decomposed using Ensemble Empirical Mode Decomposition (EEMD). IMF components were filtered using dual criteria—Hurst exponents and information entropy—effectively separating noise from valid signals. This significantly enhanced the stability and predictability of the input data. Simultaneously, to quantify the impact of media sentiment, this study developed a news sentiment mining model by constructing a financial sentiment lexicon and extending it based on the mutual information (MI) algorithm. This model calculates the daily sentiment polarity index BI, which is introduced into the predictive model as a structured market sentiment variable. In the core forecasting module, the CNN-QRLSTM model utilizes a convolutional neural network to capture the local spatio-temporal characteristics of price sequences. By incorporating a quantile regression mechanism into the LSTM, it achieves simultaneous output of point forecasts and interval forecasts. This not only provides a more comprehensive quantification of uncertainty, helping investors assess the potential scope of risks, but also overcomes the limitations of single-point forecasts in risk decision-making. To validate the framework’s reliability, we conducted a series of rigorous evaluation tests. The results indicate that this forecasting framework delivers optimal performance at a 50% confidence level and demonstrates strong resilience against risks.

Through a series of methodological innovations and empirical analyses, this study strongly proves the synergistic effect of multi-source data fusion, quantile regression, and sentiment mining and provides a two-dimensional analytical framework of “accuracy + risk” for gold price forecasting. Looking ahead, our forecasting framework can be further expanded to include high-frequency data processing, cross-market linkage analysis (e.g., a correlation study of cryptocurrencies and gold), and real-time sentiment monitoring, which is expected to further promote the widespread application of quantitative forecasting models in complex financial markets and help financial market participants grasp the opportunities in the midst of uncertainty.

## Figures and Tables

**Table 1:** Literature Review on Factors Affecting Gold Prices.

Author	Factor	Research Methodology	Conclusion
Huang (2010) [7]	US dollar index	-	There is a negative correlation between the price of gold and the US dollar index, and there is no cointegration relationship.
Xie (2010) [8]	US dollar index	Cointegration equation	There is a little bit of cointegration between the world gold price and the US dollar.
Dan (2009) [9]	CPI	The Phillips expanding curve equation and the method of least squares estimation	The price of gold plays a role in forecasting the inflation, and can be used as a reference to indicate the economic trends and the changes of the inflation fluctuations.
Zhu (2018) [10]	Monetary policy	-	The Federal Reserve and the European Central Bank have had a large impact on the gold price, while the monetary policies of the Bank of England and the Bank of Japan have had no significant impact on the gold price.
Zhou (2025) [11]	Geopolitical risk	The log-periodic power law singularity (LPPLS) model	A significant relationship between GPR and gold price bubbles, particularly with GPRA, which exerts a stronger influence than GPRT does.
Apergis (2019) [12]	Effective interest rate	VECM	There is a positive correlation between the price of gold and real interest rates.
Kanjilal (2017) [13]	Crude oil	Dual-mechanism threshold vector error correction Model	The relationship between gold and oil prices is nonlinear and asymmetric.
Zhang (2010) [14]	Crude oil price	-	The price of oil is positively linked to the price of gold in most cases.
Zhu (2023) [15]	Climate risk	Predictive modeling and STL decomposition	Gold price volatility is negatively correlated with physical risk.
Soni (2023) [16]	EPU	Wavelet Method	In the short to medium term, gold prices are positively correlated with EPU, but the effect is not significant; in the long term, EPU has a positive impact on gold prices.
Luo (2022) [17]	Investor sentiment	Infinite Hidden Markov (IHM) transformation model within a Heterogeneous Autoregressive (HAR)	The predictive role of investor sentiment-related factors in improving the accuracy of forecasting commodity volatility dynamics.

**Table 2:** Gold Price Forecasting Modeling Research Literature Review.

Author	Predictive Model	Research Methodology	Conclusion
Ismail (2009) [18]	Multiple Megression Model	Influencing factors such as inflation, exchange rates, and money supply	Forecast the future movement of the gold price based on the influencing factors.
Amina (2015) [19]	Bivariate vector autoregression-VAR-GARCH	Dynamic Returns and Forecasts of China’s Gold and Stock Markets	Assessed the diversification and hedging effectiveness of gold in China.
Li (2024) [20]	Combining BP neural networks and ensemble empirical modal decomposition to build a new model	-	The effect of white noise is reduced compared to the original BP neural network to improve the robustness of the model.
Hadavandi (2010) [21]	Particle swarm optimization (PSO)	Parameter estimation using PSO algorithm	It is able to cope with the volatility of the gold price time series and has good prediction accuracy.
You (2025) [22]	CNN and LSTM	-	Extracting data features to improve prediction accuracy.
Poor (2024) [4]	Introducing unstructured data to construct a CNN-based gold price prediction model	-	A highly accurate decision support tool for investors and financial institutions.
Solikhun (2025) [23]	Quantum computer	-	Multiple computations can be performed simultaneously, enabling the solution of problems that are difficult to solve with classical computers.
Nallamothul (2024) [24]	SKGARCH and LSTM for skewness and Kurtosis	-	The problem of under-consideration of volatility information and non-normal distribution characteristics in traditional methods is addressed.
Guo (2025) [25]	The VMD-RES.-CEEMDAN-WOA-XGBoost model	Variable selection using a traditional LASSO model followed by prediction using QRNN	CEEMDAN is employed to decompose a residual term containing complex information following the VMD and XGBoost optimized by the WOA
Bhavana (2025) [26]	A multi-objective optimization framework	Utilizes Pareto alpha cutting techniques to evaluate and enhance gold price forecasting models	ARDL achieves excellent accuracy and goodness-of-fit, while the stochastic model exhibits robust stability.
Gijy (2025) [27]	MWFKTS-RPWO	-	It provides an optimal balance between computational efficiency and accuracy compared to existing methods.
Wu (2025) [28]	An improved brain-inspired neural network	The GELU function and residual connections	By utilizing residual connections to transmit information between shallow and deep layers, the network fully leverages information to mine deep hidden features.
Chen (2025) [29]	DROI framework	Coupled with an econometric breakpoint test	Effectively addressed the inherent complexities of electricity prices.
Che (2025) [30]	CEEMDAN-GAFSF-DBiGRU-OLSSA	-	It employs multi-temporal and spatial characteristics for wind speed modeling to enhance information acquisition and complex pattern analysis.

**Table 3:** Vocabulary of financial terms and word formation in the Basic Affective Dictionary (partial).

Financial Words	Positive Emotional Words	Positive Evaluation Words	Negative Emotion Words	Negative Evaluation Words
developmental	carry	peaceful	worried	exorbitant
governments	praise	insurance	pessimism	conservative
economics	reverence	indispensable	tentative	complicated
market	favor	impartial	muffled	monotonous
resource	complacent	well-to-do	bad reaction	take time
offerings	gratitude	fairness	awkward	high cost
interest	solicitous	convenient	anxiety	dim
buying	exuberant	reliably	panic-stricken	cunning
odds	thirst	full of vitality	attack	straitened circumstances

**Table 4:** Word formation of auxiliary dictionaries (partial).

Degate Words	Stop-Word Phrases	Degree Level Terms
not yet	including	very
unavoidable	and	especially
not	in order to	a little bit
difficult	what	mildly
why bother	thereby	counterpart
none	but	more and more
nothing	for example	adequately
less	in addition	a little
un-	also	too

**Table 5:** Classification of influencing factors.

Form	Factor	Digital Indicators
Macroeconomic	US dollar index	USDX
	Inflation	CPI
	Effective interest rate	EIR
Economic and Political	Monetary policy	M2
	Economic policy	EPU
Others	Bulk commodities	Petroleum
		Copper
	Climate risk	ACI
	Media sentiment	BI

**Table 6:** Hurst value of IMF after decomposition.

IMF	WGC	LBMA	SGE
IMF1	−0.004	−0.002	−0.005
IMF2	−0.016	−0.015	−0.013
IMF3	0.012	0.005	0.020
IMF4	0.133	0.131	0.140
IMF5	0.575	0.400	0.534
IMF6	0.915	0.844	0.867
IMF7	0.995	0.984	0.999
IMF8	0.974	0.975	0.972

**Table 7:** Seed Word Collection (partial).

Positive Seed Words	Negative Seed Words
appreciation	price reduction
firm	tumble
steady	fall apar
favorable	negative
buy	sell
signal	diving
peace party	war party
praise	weak

**Table 8:** Golden Dictionary of Emotions (partial).

Positive Emotional Words	Negative Emotion Words
rising significantly	stresses
escalate	price reduction
rising	sell
steady	depreciation
loose	disadvantageous
look forward to	damages
open	slump
work force	panic-stricken
deterministic	bullion rush
flood in	deeply entrenched

**Table 9:** WGC Combined performance across assessment indicators.

BI	Model	MAE	MSE	RMSE	MAPE	R^2^
Without BI data	LSTM	26.505	1225.715	35.010	1.254	0.989
	CNN-LSTM	39.061	4382.585	66.201	1.794	0.957
	CNN-QRLSTM	31.751	1504.485	38.788	1.543	0.986
	EEMD-CNN-LSTM	27.079	1271.598	35.660	1.123	0.988
	EEMD-CNN-QRLSTM	21.770	1043.686	32.306	0.874	0.990
With BI	LSTM	20.670	743.864	27.274	0.974	0.993
	CNN-LSTM	26.761	1240.784	35.225	1.262	0.987
	CNN-QRLSTM	32.300	965.040	31.065	1.201	0.991
	EEMD-CNN-LSTM	20.174	1210.872	34.780	0.844	0.989
	EEMD-CNN-QRLSTM	**13.200**	**281.660**	**16.783**	**0.542**	**0.998**
	XGBoost	312.880	106,787.051	326.783	18.244	−11.169
	ARIMA-GRACH	224.997	60,157.020	245.269	13.573	−5.855

**Table 10:** LBMA Combined performance across assessment indicators.

BI	Model	MAE	MSE	RMSE	MAPE	R^2^
Without BI data	LSTM	23.487	1077.188	32.821	1.218	0.987
	CNN-LSTM	28.941	1715.414	41.417	1.468	0.981
	CNN-QRLSTM	27.436	2521.761	50.217	1.400	0.972
	EEMD-CNN-LSTM	31.049	2547.504	50.473	1.582	0.968
	EEMD-CNN-QRLSTM	16.002	515.246	22.699	0.871	0.993
With BI	LSTM	13.652	467.667	21.626	0.709	0.995
	CNN-LSTM	19.791	812.582	28.506	1.300	0.990
	CNN-QRLSTM	15.820	538.650	23.209	0.807	0.993
	EEMD-CNN-LSTM	16.362	470.668	21.695	0.848	0.994
	EEMD-CNN-QRLSTM	**10.117**	**167.209**	**12.931**	**0.535**	**0.998**
	XGBoost	312.880	102,845.783	320.695	17.683	−10.874
	ARIMA-GRACH	218.654	58,243.876	241.338	12.875	−5.630

**Table 11:** SGE Combined performance across assessment indicators.

BI	Model	MAE	MSE	RMSE	MAPE	R^2^
Without BI data	LSTM	26.505	1225.715	35.010	1.254	0.989
	CNN-LSTM	39.061	4382.585	66.201	1.794	0.957
	CNN-QRLSTM	32.951	2009.762	44.830	1.506	0.986
	EEMD-CNN-LSTM	30.894	2105.213	45.883	1.362	0.985
	EEMD-CNN-QRLSTM	21.083	1137.877	33.732	0.894	0.992
With BI	LSTM	28.172	1469.841	38.339	1.300	0.992
	CNN-LSTM	34.111	2504.921	50.049	1.513	0.985
	CNN-QRLSTM	24.611	965.040	31.065	1.201	0.991
	EEMD-CNN-LSTM	23.875	1223.978	34.985	1.056	0.992
	EEMD-CNN-QRLSTM	**21.590**	**1054.347**	**32.470**	**0.952**	**0.993**
	XGBoost	289.457	91,267.845	302.105	16.894	−9.765
	ARIMA-GRACH	198.765	45,892.768	241.338	11.987	−4.987

**Table 12:** Extreme Condition Performance.

Market	Market Scenario	RMSE	MAE	R^2^
WGC	Normal Stability	13.520	10.480	0.999
	High-Volatility Rally	19.240	15.120	0.997
	High-Volatility Drop	23.460	18.350	0.995
LBMA	Normal Stability	29.830	22.680	0.995
	High-Volatility Rally	37.240	29.150	0.992
	High-Volatility Drop	41.810	33.050	0.990
SGE	Normal Stability	10.760	8.390	0.999
	High-Volatility Rally	16.310	12.790	0.998
	High-Volatility Drop	19.390	15.210	0.996

## Data Availability

This paper provides gold price data from the WGC, LBMA, and SGC markets used in the model.
